# The comparison of cytotoxic and genotoxic activities of glucosinolates, isothiocyanates, and indoles

**DOI:** 10.1038/s41598-022-08893-8

**Published:** 2022-03-22

**Authors:** Dominik Kołodziejski, Izabela Koss-Mikołajczyk, Hansruedi Glatt, Agnieszka Bartoszek

**Affiliations:** 1 Department of Food Chemistry, Technology and Biotechnology, Gdansk University of Technology, Narutowicza St. 11/12, 80-233 Gdansk, Poland; 2 Department of Nutritional Toxicology, German Institute of Human Nutrition, Potsdam-Rehbrücke, 14558 Nuthetal, Germany

**Keywords:** Cell culture, DNA, Chemical modification

## Abstract

Chemopreventive properties of Brassica vegetables are attributed mainly to their characteristic compounds—glucosinolates (GLs) and their main hydrolysis products—isothiocyanates (ITCs) and indoles. In this study, we compared antiproliferative activity (MTT test in HT29 cells) and genotoxic effects (comet assay in HT29 cells and restriction analysis in a cell-free system) of three GLs (sinigrin (SIN), glucotropaeolin (GTL), and glucobrassicin (GLB)) with that of their major degradation products. Intact GLs did not exhibit cytotoxic activity, possibly due to their limited bioavailability. However, in the presence of myrosinase (MYR), GLs gained the ability to inhibit HT29 cells’ growth. The addition of MYR caused the hydrolysis of GLs to the corresponding ITCs or indoles, i.e. compounds that show stronger biological activity than parent GLs. Pure ITC/indole solutions showed the strongest antiproliferative activity. Based on the results of restriction analysis, it was found that GLs to a greater extent than ITCs caused DNA modification in a cell-free system. In the case of GLs, metabolic activation by the S9 fraction increased this effect, and at the same time changed the preferential binding site from the area of base pairs AT to GC base pairs. Of all compounds tested, only benzyl ITC caused DNA damage detectable in the comet assay, but it required relatively high concentrations.

## Introduction

Over the past three decades, numerous studies have proved that increased consumption of the *Brassicaceae* family vegetables may significantly reduce the risk of so-called civilization diseases, such as cancer^1^ cardiovascular ailments^2^, neurodegenerative diseases^3^ and type 2 diabetes^4^. Chemopreventive properties of cruciferous vegetables are ascribed to the characteristic secondary metabolites—glucosinolates (GLs). It is widely believed that GLs do not exhibit direct biological activity*;* it is attributed to some of their hydrolysis products released by the enzyme myrosinase (MYR) and associated proteins^5^, among which isothiocyanates (ITCs) and indoles are the most biologically active^6^. It must be emphasized, however, that all health claims prioritizing ITCs and indoles over GLs or other GL hydrolysis products are based mainly on investigations performed with only a handful of selected derivatives. Though these shortcomings are well understood, in literature the dominant approach relies on either the use of plant extracts in which the content of ITCs and indoles is estimated based on the initial content of endogenous GLs or commercially available degradation products are applied. However, some data illustrating the chemopreventive potential of intact GLs can be found in the scientific literature^7–10^. Canistro et al.^7^ investigated the ability of GL—gluconasturtin (GNST) to modulate the activity of xenobiotic-metabolizing enzymes in male Swiss albino CD1 mice injected with single or multiple doses of GNST. The results suggested that GNST strongly inhibited the enzymes of phase I xenobiotic metabolism. Perocco et al.^8^ showed that another GL—glucoraphanin (GRE) induced both phase I and phase II detoxification enzymes. In the study of Barillari et al.^9^, male Sprague–Dawley rats were treated with glucoraphasatin (GRH) or glucoraphanin (GRE), in single or repeated doses. After treatment, the hepatic microsomal fraction was prepared, and xenobiotic-metabolizing enzymatic activities were analyzed. Phase I and phase II metabolizing enzymes were significantly induced by both tested GLs.

Another aspect of the GLs vs. ITCs bioactivity debate pointed by some researchers is the fact that if MYR is inactivated by cooking process, intact GLs pass to the colon, where they are degraded by colonic microbiome^11^. This implies, that humans are hardly ever exposed to ITCs by dietary consumption of Brassica vegetables and, only at most one-third of the ingested GLs are converted to ITCs by intestinal thioglucosidases^12^. Despite this, most reports refer to in vivo studies on ITCs instead of GLs^13^, overlooking the fact that GLs are the compounds naturally occurring in the diet.

The above review summarizing the so far research on the bioactivity of intact GLs and no published reports comparing the biological properties of GLs and products of their hydrolysis made us undertake such an initial systematic comparison of these food components’ impact on human colon adenocarcinoma (HT29) cells.

**Figure 1:**
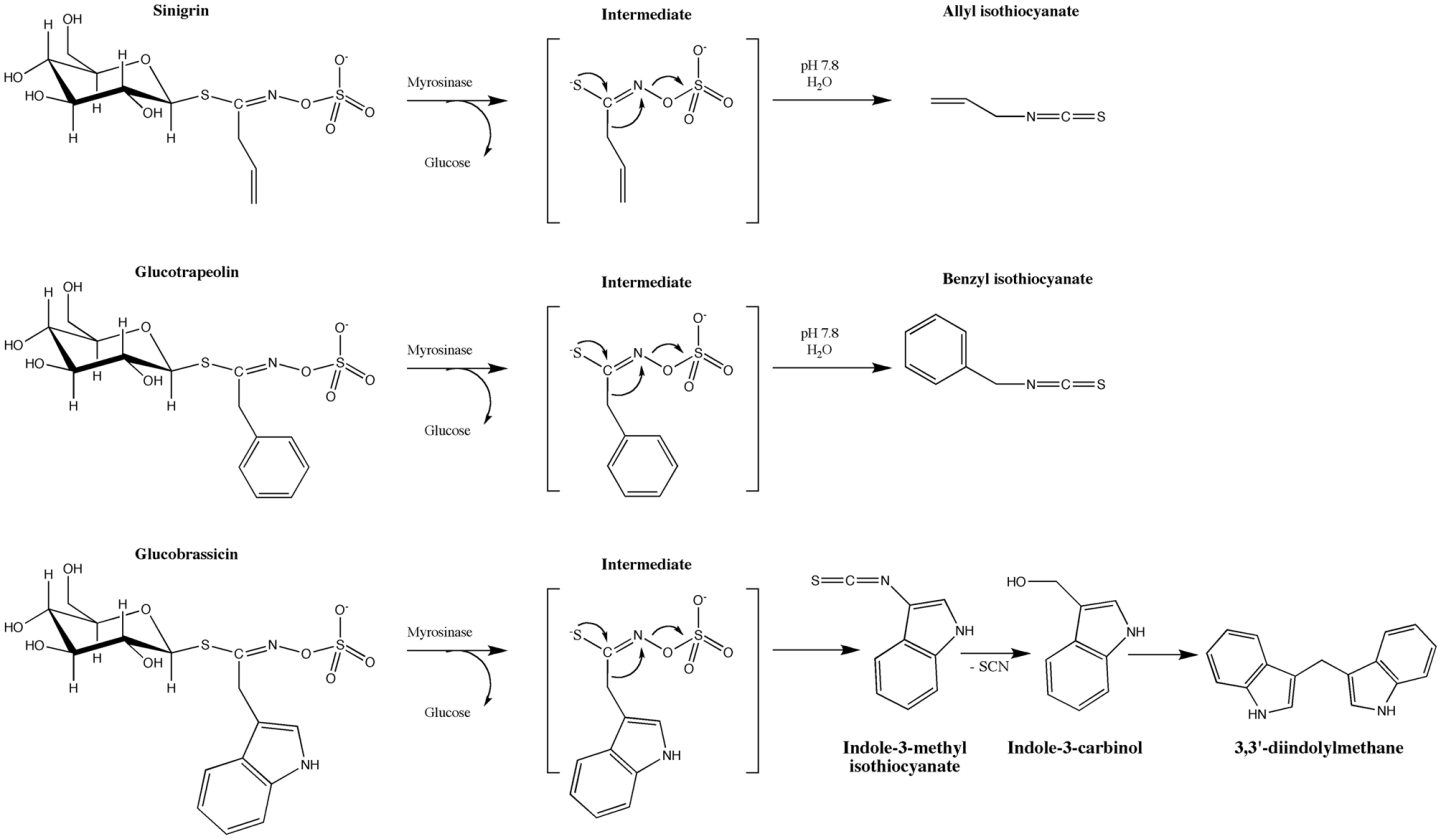
Glucosinolates studied and their degradation products.

The scope of the study embraces antiproliferative (MTT test) and genotoxic (comet assay and restriction analysis) properties of three representative GLs and corresponding best-characterized degradation products liberated upon MYR action. The tested substances included (Fig. 1): intact GLs with aliphatic (sinigrin—SIN), aromatic (glucotropaeolin—GTL) or indolic (glucobrassicin—GLB) side chain as well as their major hydrolysis products, allyl isothiocyanate (AITC), benzyl isothiocyanate (BITC) and indole-3-carbinol (I3C), respectively. In addition, the neoglucobrassicin decay product—*N*-methoxy-indole-3-carbinol (NI3C) and the indole-3-carbinol dimerization product—3,3′-diindolylmenthane (DIM) were evaluated. The choice of the latter compounds was dictated by literature reports on their genotoxic and mutagenic effects reviewed by Latté et al.^14^.

## Results and discussion

### Antiproliferative activity of GLs and their hydrolysis products

**Figure 2:**
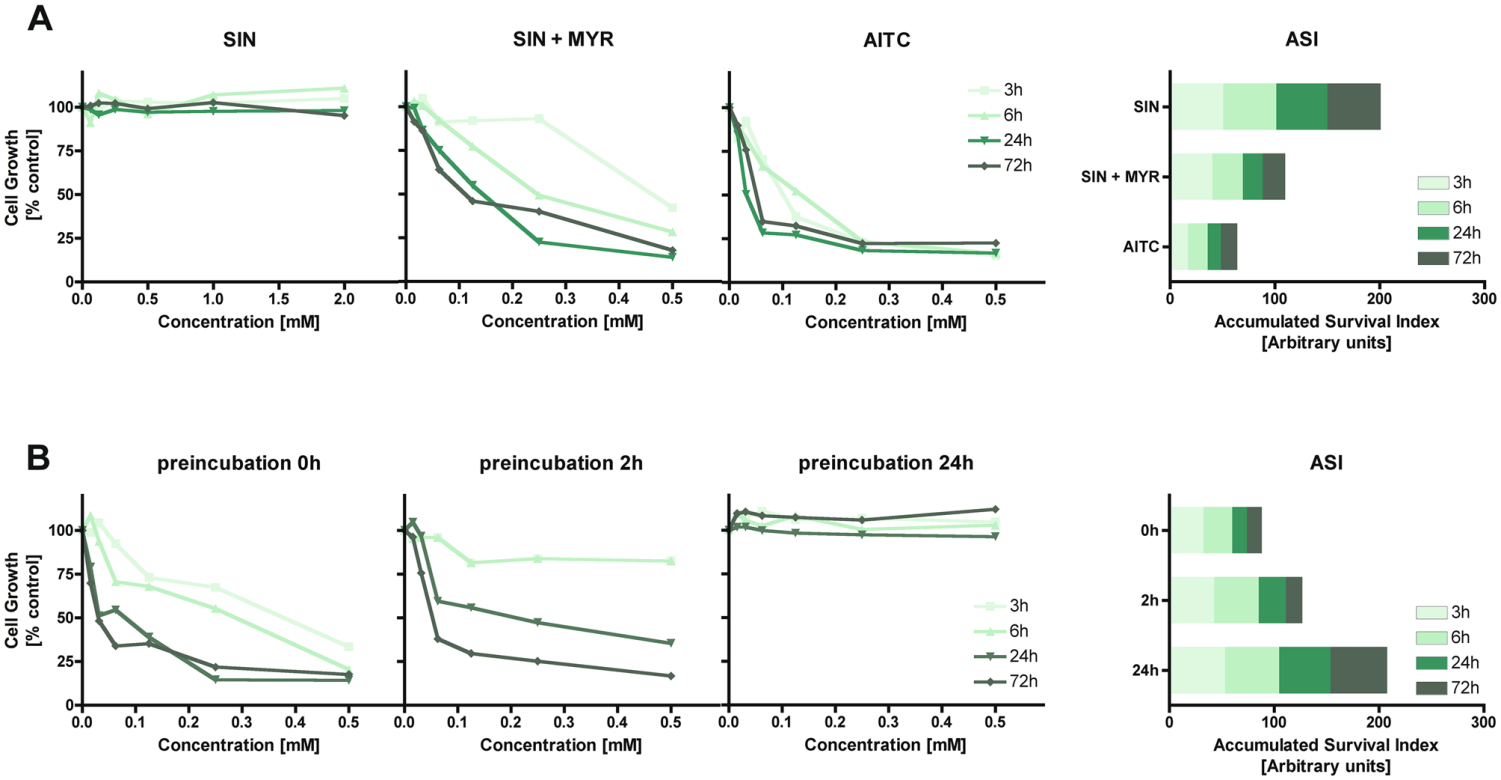
Growth inhibition of human adenocarcinoma cancer cells (HT29) treated with GL or its derivative directly (**A**) or after preincubation with myrosinase (**B**) evaluated by MTT test after different incubation periods (3, 6, 24, and 72 h). (**A**) Cells exposed to sinigrin (SIN), sinigrin, and myrosinase (SIN + MYR) or SIN hydrolysis product—allyl isothiocyanate (AITC). (**B**) Cells exposed to the combination of SIN and MYR incubated before cell treatment for different periods (0, 2, and 24 h). Accumulated Survival Index (ASI) is defined as the sum of areas under survival curves determined for individual treatments for the same concentration range (0–0.5 mM). The results represent the means of three independent experiments; SD values did not exceed 15%.

**Figure 3:**
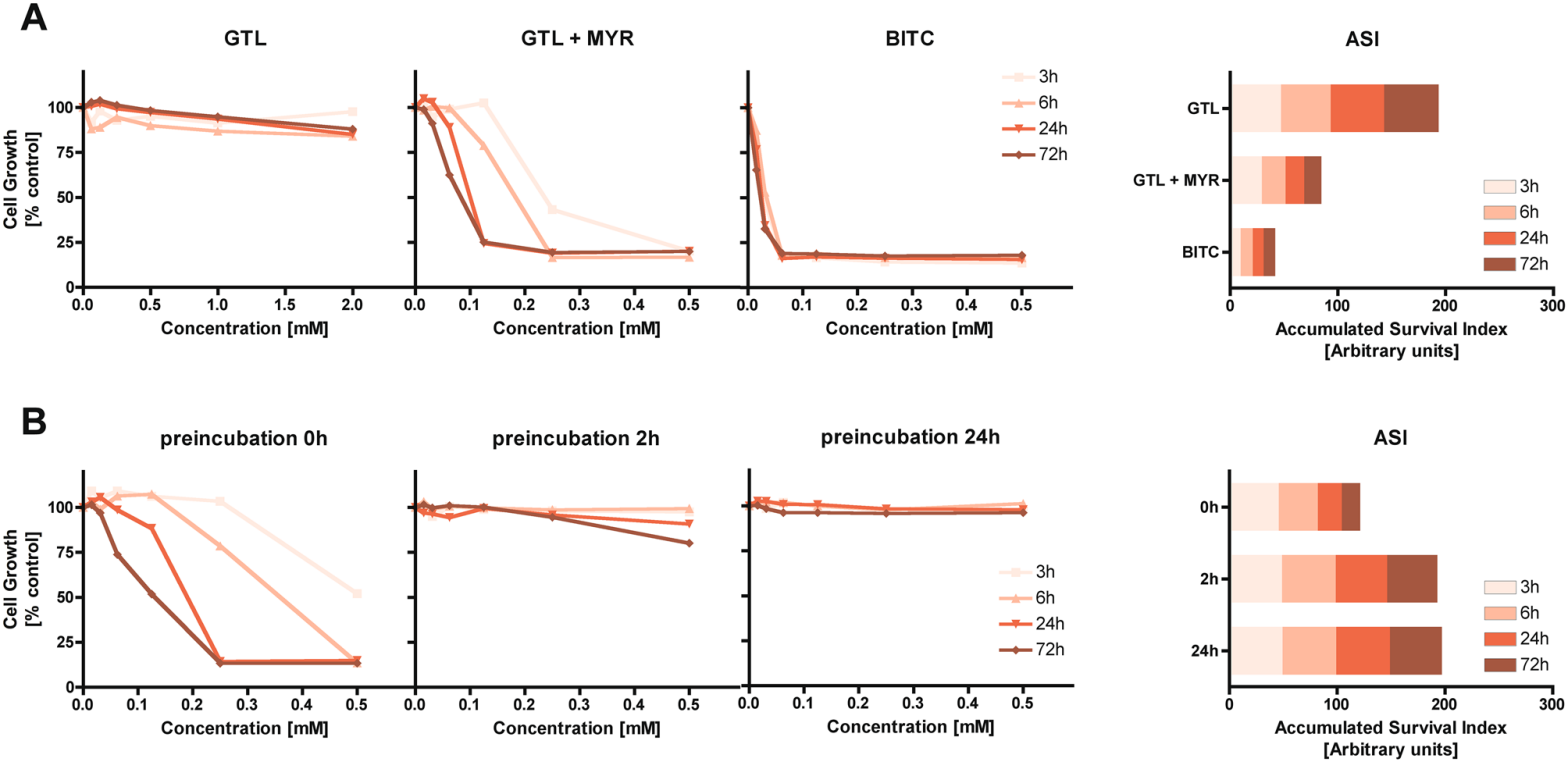
Growth inhibition of human adenocarcinoma cancer cells (HT29) treated with GL or its derivative directly (**A**) or after preincubation with myrosinase (**B**) evaluated by MTT test after different incubation periods (3, 6, 24, and 72 h). (**A**) Cells exposed to glucotropaeolin (GTL), glucotropaeolin, and myrosinase (GTL + MYR) or GTL hydrolysis product—benzyl isothiocyanate (BITC). (**B**) Cells exposed to the combination of GTL and MYR incubated before cell treatment for different periods (0, 2, and 24 h). Accumulated Survival Index (ASI) is defined as the sum of areas under survival curves determined for individual treatments for the same concentration range (0–0.5 mM). The results represent the means of three independent experiments; SD values did not exceed 15%.

**Figure 4:**
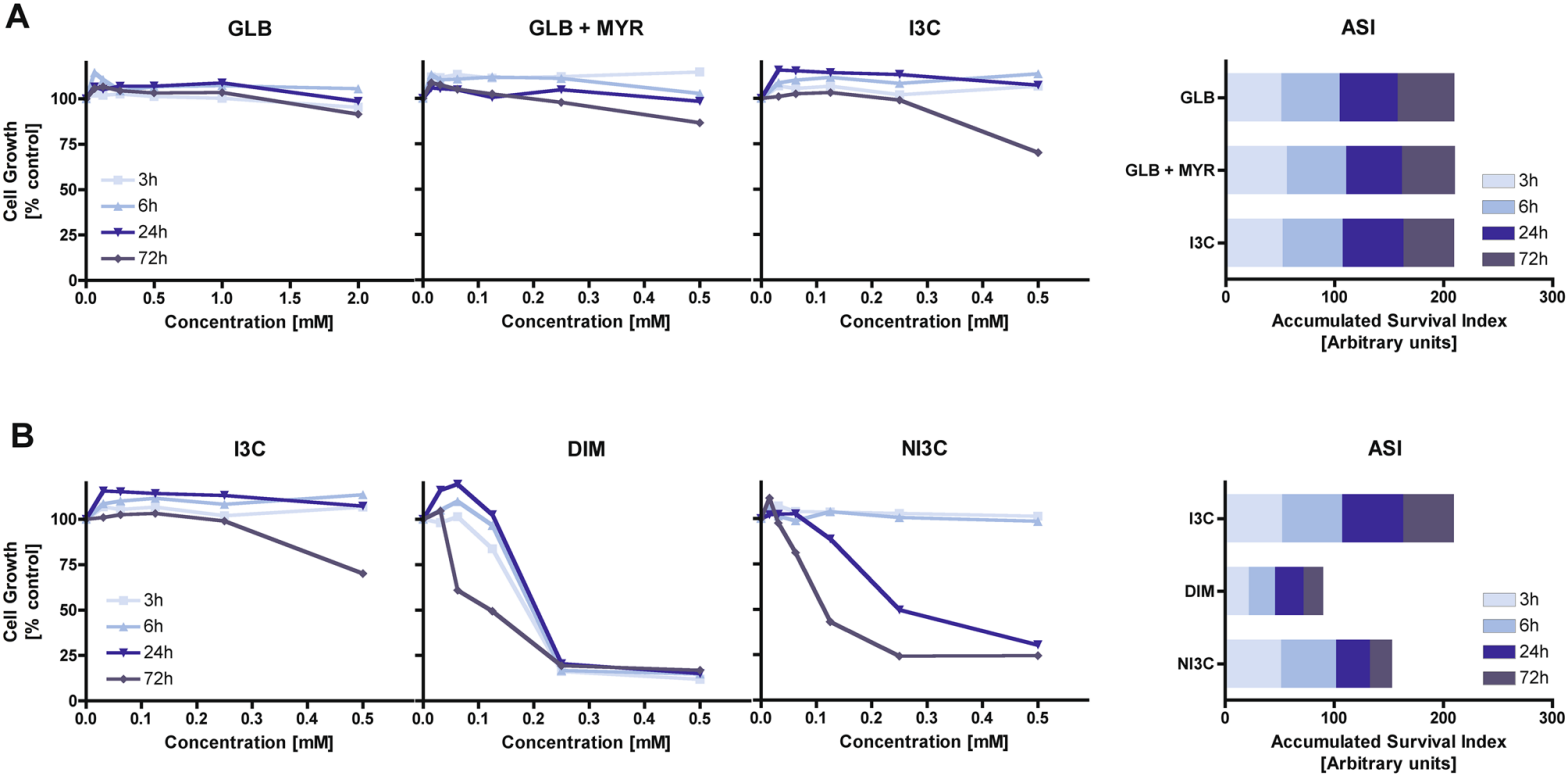
Growth inhibition of human colon adenocarcinoma cancer cells (HT29) treated with GL or its derivatives evaluated by MTT test after different incubation periods (3, 6, 24, and 72 h). (**A**) Cells exposed to glucobrassicin (GLB), glucobrassicin, and myrosinase (GLB + MYR) or GLB hydrolysis product—indole-3-carbinol (I3C). (**B**) Comparison of cell growth inhibition caused by I3C, its dimer DIM or *N*-methoxy-indole-3-carbinol (NI3C). Accumulated Survival Index (ASI) is defined as the sum of areas under survival curves determined for individual treatments for the same concentration range (0–0.5 mM). The results represent the means of three independent experiments; SD values did not exceed 15%.

The ability to inhibit the growth of cultured cancer cells by Brassicaceae vegetables has been documented before in several cell lines in vitro^10,15–18^. Antiproliferative activity of these vegetables is assigned to the presence of GLs often assuming their equivalence with that of their hydrolysis products, in particular of ITCs and indoles. The investigations comparing the activity of parent GLs with that of their degradation products are very scarce. In this study, we concentrated on such comparisons using colon cells as a model of gastrointestinal tract directly exposed to both kinds of these phytochemicals. Specifically, we selected human colon adenocarcinoma HT29 cells to play a dual role. On one hand, this cell line is recommended for nutritional studies^19^ to mimic alimentary tract epithelium, on the other hand, these are transformed cells, hence may indicate the anticarcinogenic effects of tested substances. To compare the antiproliferative activity of GLs with that of their most active hydrolysis products as well as the impact of myrosinase on GLs activity, cells were treated for 3, 6, 24, and 72 h with solutions of tested GLs, GLs in the presence of myrosinase (GLs + MYR) or corresponding ITCs/indoles. The cell growth was determined using the MTT test. The results are presented in Figs. 2, 3, 4 as survival curves and as Accumulated Survival Index (ASI) calculated according to the description given in “Materials and methods” (“Antiproliferative activity (MTT test)”).

#### Antiproliferative activity of an aliphatic GL—sinigrin (SIN) and its hydrolysis product—allyl isothiocyanate (AITC)

The first tested pair of GL/hydrolysis products was SIN and its derivative—AITC released as a result of MYR catalyzed reaction. AITC belongs to the most potent biocidal ITCs with documented ability to damage biomolecules of plant-attacking pathogens^20^, but also exhibiting several chemopreventive activities in human cells^21^. As shown in Fig. 2A, SIN did not influence the growth of HT29 cells regardless of treatment. In contrast, concentration- and time-dependent growth inhibition was observed when SIN was applied together with MYR (1 mU of MYR per 1 nmol of GL). Treatment of HT29 cells with SIN + MYR combination resulted in a significant increase of antiproliferative effect; ASI parameter decreased by almost twofold compared to SIN applied alone. The exposure to purified AITC solutions resulted in the strongest growth inhibition of HT29 cells after 3 h of incubation, and, as indicated by the ASI parameter, proliferation reached its lowest level following 24 h treatment (Fig. 2A, bar graph). The smaller antiproliferative effect of the SIN + MYR combination for short incubation times compared to equimolar concentrations of AITC was probably caused by its only partial release. For prolonged incubations, enabling complete hydrolysis of SIN, the inhibitory effect almost equalized with that of purified AITC. These results confirm that AITC is a major antiproliferative factor derived from SIN because, in the absence of specifier proteins that may specifically redirect the rearrangements of MYR released intermediate thiohydroximate-*O*-sulfonate to nitrile, epithionitrile, or thiocyanate, spontaneous rearrangement leads to the formation of AITC as a major hydrolysis product^5^.

The experiments in which SIN was preincubated with MYR before application to cell cultures (Fig. 2B) revealed the instability of released derivative(s). In the case of preincubation as short as 2 h, the ASI parameter increased by about 50% compared to 0 h preincubation. After 24 h preincubation, the SIN + MYR reaction mixture no longer exhibited any growth inhibitory activity when applied to HT29 cells. The possible explanation for this observation is that ITCs formed during preincubation being not very stable compounds^22^ and in the aqueous environment they could undergo further chemical conversions to derivatives that did not inhibit the growth of HT29 cells (e.g., allyl thiocyanate, allylamine, allyl dithiocarbamate, diallylthiourea, carbon disulfide, diallylurea, diallylsulfide).

#### Antiproliferative activity of an aromatic GL—glucotropaeolin (GTL) and its hydrolysis product—benzyl isothiocyanate (BITC)

The second tested pair of GL/hydrolysis products was GTL and BITC. Similarly, to previously described AITC, also BITC displays documented chemopreventive potential^23^. MTT test results (Fig. 3A) showed that alike SIN, GTL did not influence the growth of HT29 cells regardless of treatment, while concentration- and time-dependent growth inhibition was observed when GTL was applied together with MYR. Hydrolysis of GTL by MYR increased significantly the antiproliferative effect of the mixture; ASI parameter decreased more than twofold compared to GTL applied alone. The solution of purified BITC showed the strongest antiproliferative effect, which reached 80% cell growth inhibition as soon as after 3 h of incubation with 62.5 μM BITC solution. In the case of AITC, a similar effect was obtained at a much higher concentration of 250 μM. Similarly, to SIN/AITC, the lower antiproliferative effect of the GTL + MYR combination for short incubation times compared to equimolar concentrations of BITC was probably caused by the delayed release of the latter.

The effect of preincubation of GTL with MYR before cell treatment resembled also that observed for SIN (Fig. 3B). It was found that along with the prolongation of preincubation time, the antiproliferative effect towards HT29 cells decreased. This activity dropped to an even much greater extent than in the corresponding experiments with SIN. After 2 h preincubation, the GTL + MYR reaction mixture no longer exhibited any growth inhibitory activity when applied to HT29 cells, while in the case of SIN/MYR this happened after 24 h preincubation. These results suggest that BITC is even more unstable than AITC under tested conditions and undergoes some further chemical changes during the preincubation period. Our observations are in accord with the degradation of BITC described earlier where the formation of benzylamine was determined^24^.

#### Antiproliferative activity of indole GL—glucobrassicin (GLB) and its hydrolysis products—indole-3-carbinol (I3C), and a congener—*N*-methoxy-indole-3-carbinol N(I3C)

The third tested pair of GL/hydrolysis product was GLB and its derivative**—**I3C (Fig. 4A). GLB contains the indole side chain, which in the presence of MYR initially transforms mainly to ITCs and nitriles depending on the reaction environment (Fig. 1). However, due to the low stability of indole ITCs, they undergo further rapid rearrangements: initially to alcohol, in the case of GLB to I3C^25^. For GLB, the concentration range used was lower than for SIN and GTL due to limitations in its availability. Additionally, the antiproliferative activity of I3C was compared with that of another indole GL—neoglucobrassicin (neoGLB) derivative—*N*-methoxy-indole-3-carbinol (NI3C) (Fig. 4B) known for its high toxicity^26^.

MTT test results showed that similar to the other tested GLs (SIN and GTL), GLB alone did not influence the growth of HT29 cells regardless of treatment (Fig. 3A). However, in contrast to SIN and GTL, the combination of MYR and GLB did not lead to an antiproliferative effect either. The main purified breakdown product I3C caused only 30% growth inhibition, at the highest concentration (500 μM) and the longest 72 h incubation. In comparison with AITC and BITC, the influence of I3C on growth inhibition of HT29 cells was thus less prominent. However, the methoxylated derivative NI3C at longer incubation times (24 h and 72 h) exhibited antiproliferative activity comparable with that of AITC (Fig. 3B). The increase in antiproliferative effect only after long incubation periods may indicate a different mechanism of action of indole derivatives compared to ITCs. NI3C is known to induce lesions at the DNA level^26^, which may be effectively removed at low concentrations by the action of DNA repair mechanisms. Only the accumulation of such events leads to cytotoxicity, therefore it is observed after prolonged exposures of cells.

The results demonstrate that GLs are rather inert biologically compounds and probably too hydrophilic to pass cellular membranes and to become bioavailable inside cells where they might be deglucosylated by intracellular glycosidases. Lipophilic low-molecular-weight ITCs can easily penetrate cell membranes, become effectively biodistributed, and initiate biological processes e.g., formation of dithiocarbamates with cysteines present in proteins^27^, leading to the blockage of proliferation. The antiproliferative action of indolic derivatives seems to be more structure-dependent. Accordingly, the growth inhibitory properties of I3C that may be commonly present in vegetables are not very strong, while effects of NI3C known for its toxicity are matching those of ITCs for prolonged incubations.

If these findings were interpreted in the context of consumption of *Brassica* vegetables as a gastrointestinal cancer-preventive ingredient, the obvious recommendation would be their intake in a fresh form as rather big chunks, so the MYR released ITCs are generated in situ in the alimentary tract. It seems that “ready-to-use” chopped salads may be substantially less rich with bioactives. Cooking, especially prolonged, will cause inactivation of MYR, thus in this case the release of ITCs is believed to be dependent on the composition of the consumer’s microbiome^28^, however, Budnowski et al.^29^ indicated that the influence of intestinal bacteria on the degradation of GLs is rather limited. These conflicting results show that more research is needed to identify the role of the microbiome in GLs hydrolysis.

### The ability of GLs and their hydrolysis products to induce DNA fragmentation

The comet assay was used to determine the ability of GLs and their hydrolysis products to cause DNA fragmentation in individual eukaryotic cells. HT29 cells were treated with tested solutions of GLs, GLs in the presence of MYR, and corresponding ITCs/indoles. Following overnight exposure of the cells, a comet assay was performed. This experimental approach enabled not only to observe the ability of GLs and their breakdown products to cause DNA fragmentation but also to reason about the persistence of this DNA damage since the prolonged exposure enabled cellular DNA repair mechanisms to seal the gaps in polynucleotides.

**Figure 5:**
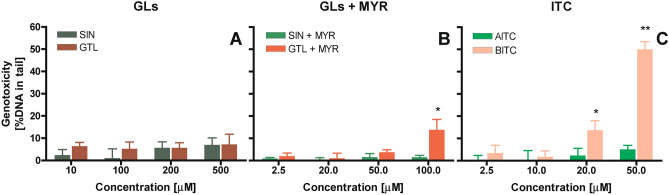
Genotoxic effect in HT29 cells determined for (**A**) glucosinolates (GLs)—sinigrin (SIN) and glucotrapeolin (GTL); (**B**) glucosinolates in the presence of myrosinase (SIN/GTL + MYR) and (**C**) released upon GLs hydrolysis isothiocyanates (ITC)—allyl isothiocyanate (AITC) and benzyl isothiocyanate (BITC). As negative control served the cells treated with pure water instead of tested compounds (± 5% DNA in tail); as positive control served the cells treated with 100 µM H_2_O_2_ (± 80% DNA in tail). The results represent means ± SD of three independent determinations. Significantly different values determined by one-way analysis of variance (ANOVA) with Dunnett’s post-test are marked as *p < 0.05, **p < 0.01.

The results showed that both SIN and GTL failed to cause persistent DNA fragmentation (Fig. 5A). DNA lesions at the level of 5% DNA in tail were in the range of negative control. These results complied with MTT test results showing that GLs themselves, probably due to their high polarity and/or limited bioavailability, exhibited low toxicity.

GLs hydrolysis by MYR caused a slight increase in the level of persistent DNA strand breaks (14% DNA in tail), but only in the case of GTL (Fig. 5B). In the case of SIN, the liberated AITC did not induce persistent DNA fragmentation. This agrees with human studies, where AITC initially caused an increase in DNA damage, which however rapidly disappeared. Therefore, it can be concluded that DNA breaks in the case of AITC are not permanent and are effectively repaired by DNA repair mechanisms. In the case of BITC, there is a strong and concentration-dependent increase in genotoxicity up to 50% DNA in the tail at the highest concentration applied (50 μM), which suggests that the lesions induced are more difficult to repair. These observations may be an explanation for why BITC exhibited stronger cytotoxicity compared to AITC.

**Figure 6:**
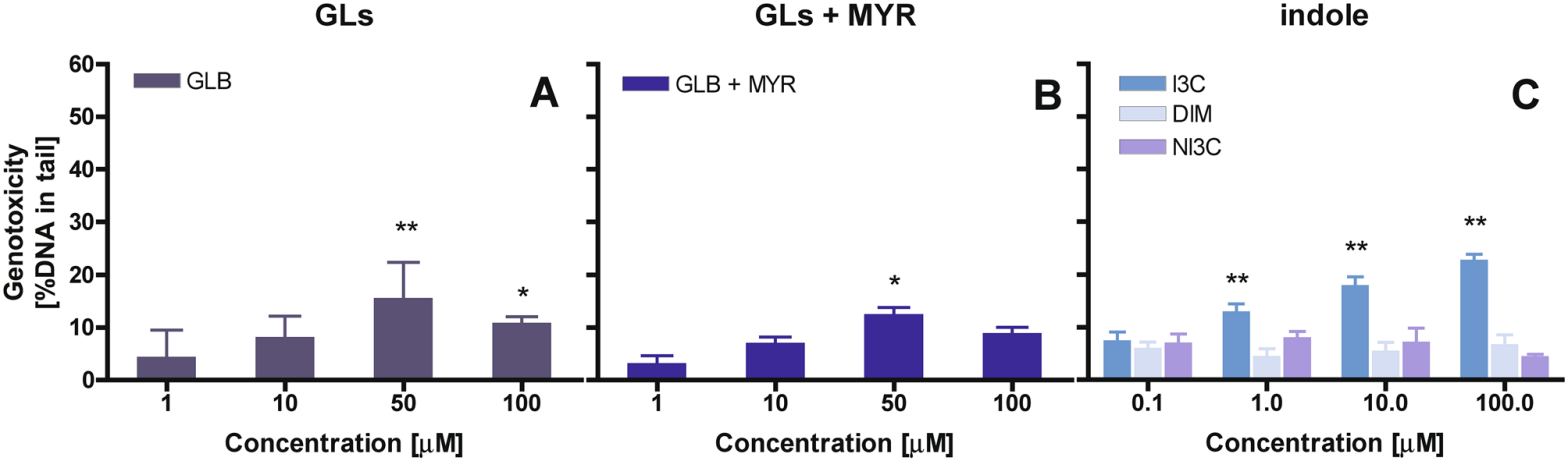
Genotoxic effect in HT29 cells determined for (**A**) glucosinolate–glucobrassicin (GLB) and GLB in the presence of myrosinase (GLB + MYR); (**B**) products of GLB hydrolysis—indole-3-carbinol (I3C), its dimer—diindolylmethane (DIM) and (**C**) neoglucobrassicin (neoGLB) hydrolysis product—*N*-methoxy-indole-3-carbinol (NI3C). As negative control served the cells treated with pure water instead of tested compounds (± 5% DNA in tail); as positive control served the cells treated with 100 µM H_2_O_2_ (± 80% DNA in tail). The results represent means ± SD of three independent determinations. Significantly different values determined by one-way analysis of variance (ANOVA) with Dunnett’s post-test are marked as *p < 0.05, **p < 0.01.

DNA persistent fragmentation induced by indolic GLB was not very strong and increased with concentration to 15% DNA in tail at a concentration of 50 μM (Fig. 6A). The presence of MYR did not affect the ability of GLB to induce DNA fragmentation (Fig. 6B).

In the case of I3C treatment, a clear dose-dependent increase in persistent DNA degradation was observed up to 20% DNA in the tail at 100 μM. Thus, it can be presumed that this indole derivative might be able to react with base pairs in cellular DNA and this persistent genotoxicity was reflected by I3C increased cytotoxicity (Fig. 4A). No persistent DNA fragmentation was found for the less toxic I3C dimerization product—DIM (Fig. 6B). The latter has been reported to exhibit chemopreventive properties such as the induction of phase I and II detoxification enzymes^10^.

Surprisingly, the most cytotoxic indole derivative NI3C, after overnight incubation failed to cause DNA fragmentation (Fig. 6), though previous studies confirmed its ability to form stable DNA adducts after activation by sulfotransferase 1A1^30^. There are two explanations of this result: HT29 cells may lack SULT1A1 activity, or the DNA adduct formed did not lead to DNA cleavage. However, the stronger cytotoxicity of this derivative in the MTT test (Fig. 4B) suggests that whatever scenario occurred, was detrimental for the cells.

### Ability of GLs and their hydrolysis products to induce covalent DNA modification in a cell-free system

To examine the ability of investigated GLs and their hydrolysis products to chemically modify DNA, the previously described restriction analysis technique was employed^31^. In this cell-free method, the DNA amplicon is incubated with a compound whose potential to form covalent bonds with nucleobases is tested and then digested with the use of either AT- or GC-specific restriction endonuclease. The inhibition of cleavage indicates chemical modification of the restriction site. Thus, the method reveals not only the formation of chemical bonds between DNA and low-molecular-weight compounds but also their base-pair preference.

#### Covalent DNA modifications by aliphatic GL (SIN) and its hydrolysis product—AITC

**Figure 7:**
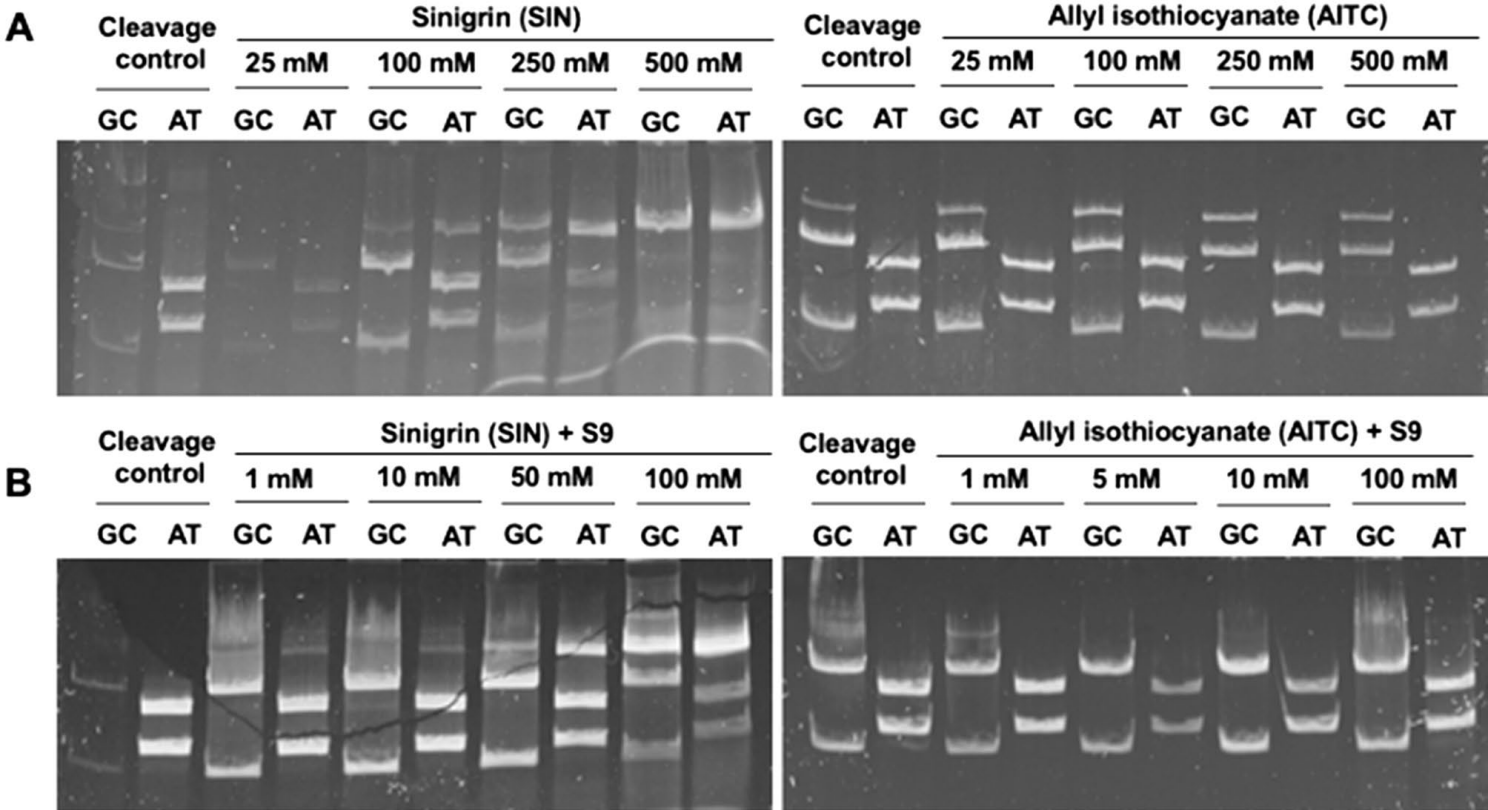
DNA fragments generated as a result of endonucleolytic cleavage of DNA amplicon with HpaII (GC-specific) or Tru1I (AT-specific) enzymes. (**A**) DNA amplicon incubated for 2 h at 37 °C with increasing sinigrin (SIN) or allyl isothiocyanate (AITC) concentrations before restriction analysis was performed as described in the “Materials and methods” section. As control served DNA amplicon incubated with water or DMSO in place of the tested compounds. (**B**) SIN and AITC were pre-incubated for 15 min at 37 °C with an S9 mixture for the metabolic activation of tested compounds. Then DNA amplicon was added to each sample and incubated for 30 min at 37 °C and restriction analysis was performed as described in the “Materials and methods” section. DNA amplicon incubated with water or DMSO in place of the test compound solution served as control.

Figure 7 shows electropherograms obtained for the amplicon incubated with SIN or AITC and then subjected to restriction analysis. The patterns obtained indicate that SIN caused DNA modification in a concentration-dependent manner. The inhibition of cleavage began above 100 mM of SIN, while at a 20-fold higher concentration, the digestion with restriction enzymes was completely inhibited. The preferential binding site of activated SIN was the AT base pair. In the case of AITC, a weak ability to modify DNA was also observed, but in GC base pairs, instead of AT, and only at a concentration above 250 mM, which is almost 2.5 times higher than in the case of SIN. The observed differences in the mechanism of binding between the GL precursor and ITC derivative suggest that other intermediate metabolites participated in the reaction with nitrogenous bases. It can be assumed that in the case of AITC, as well as other ITCs, the binding involves the isothiocyanate group for which the reaction with aromatic amines to thioureas has already been documented^10^ and with amino groups of lysine has already been documented^32^. On this basis, it is possible to predict the structures of adducts resulting from the binding of ITCs to the nitrogenous bases of DNA. More electrophilic and smaller ITCs bind to GC base pairs where two primary amino groups are present, while AT base pairs are preferentially modified by GLs.

In living organisms, the activity of GLs and their breakdown products is affected by intracellular metabolic systems, which can modify their reactivity towards DNA. Therefore, the ability to covalently modify DNA by these compounds was also tested after their metabolic activation with the S9 microsomal fraction from the rat liver, representing the cytochrome P450 system. In these experiments, concentrations of tested compounds had to be reduced, to limit the negative effect of DMSO (used to dissolve studied compounds) on the activity of the S9 fraction. Despite the relatively short incubation time (0.5 h), inhibition of DNA digestion was observed already at 1 mM SIN, while at 100 mM the digestion with restriction enzymes was strongly inhibited. It thus can be concluded that after metabolic activation, the ability of this compound to chemically modify DNA increased almost 100-fold. In addition, metabolic transformations of SIN under the influence of the S9 fraction seemed to shift the preferential binding site from AT to GC base pairs. It may be presumed that the thioglucosidic bond was hydrolyzed and that this GL spontaneously converted into intermediates with greater affinity for the amino groups of GC base pairs.

In the case of AITC, after metabolic activation by the S9 fraction, only a slight increase in the chemical DNA modification was observed, but only above 100 mM. Still, also, in this case, metabolic activation enhanced the DNA binding capacity of the compound tested. The preferential binding site of AITC has not changed, i.e., remained in GC base pairs. Thus, the amine group was still the binding site of this compound.

#### Covalent DNA modifications by aromatic GL (GTL) and its hydrolysis product—BITC

Literature data indicate that aromatic GLs and their breakdown products, to a greater extent than aliphatic ones, form DNA adducts in vitro, and show stronger mutagenicity in the Ames test^30,33^.

**Figure 8:**
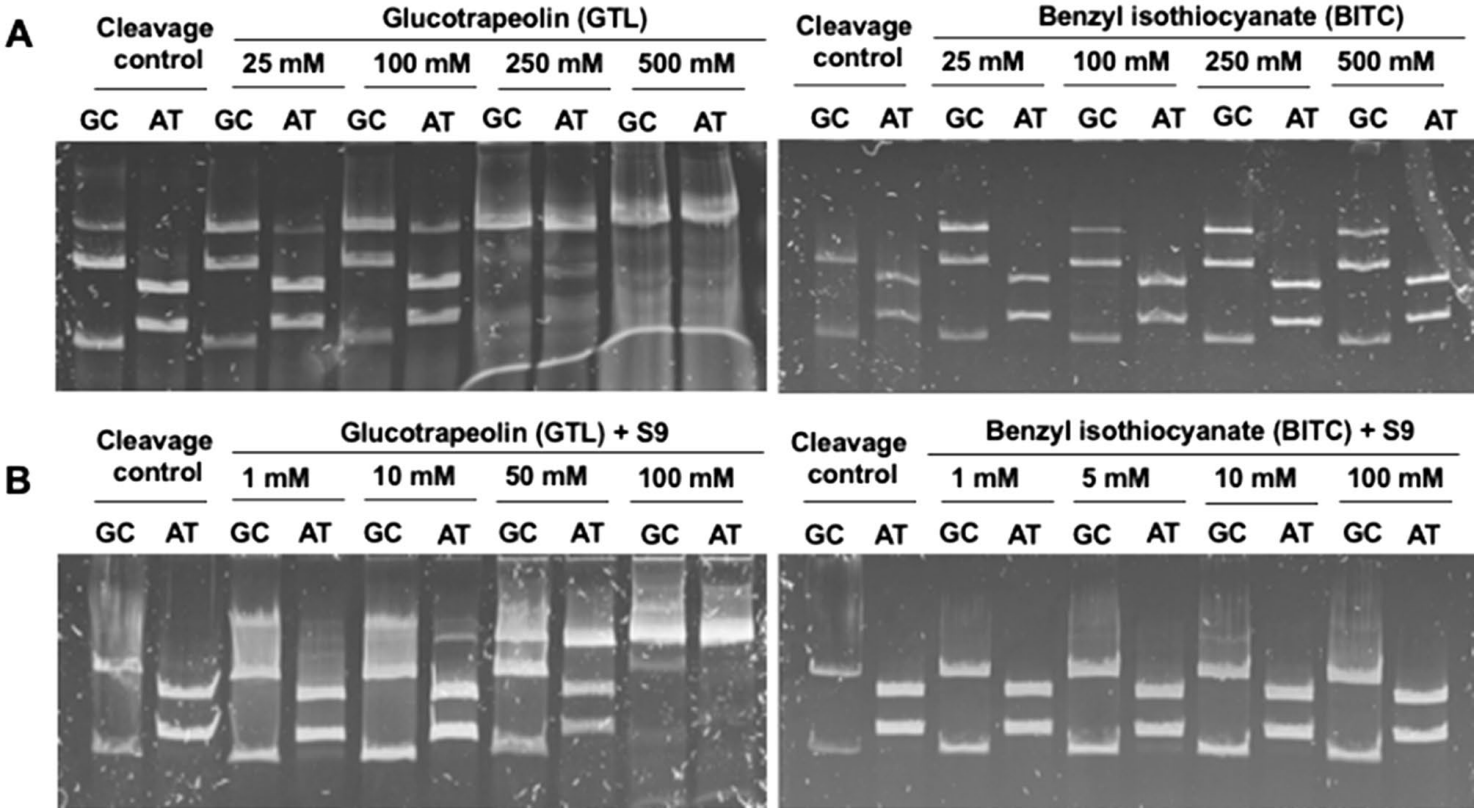
DNA fragments generated as a result of endonucleolytic cleavage of DNA amplicon with HpaII (GC-specific) or Tru1I (AT-specific) enzymes. (**A**) DNA amplicon incubated for 2 h at 37 °C with increasing glucotrapeolin (GTL) or benzyl isothiocyanate (BITC) concentrations before restriction analysis was performed as described in the “Materials and methods” section. DNA amplicon incubated with water or DMSO in place of the tested compounds served as control. (**B**) GTL and BITC were pre-incubated for 15 min at 37 °C with an S9 mixture for the metabolic activation of tested compounds. Then DNA amplicon was added to each sample and incubated for 30 min at 37 °C and restriction analysis was performed as described in the “Materials and methods” section. DNA amplicon incubated with water or DMSO in place of the test compound solution served as control.

GTL showed a concentration-dependent ability to modify DNA (Fig. 8), however, this effect was two times lower than in the case of SIN. The binding of GTL to DNA was observed from concentrations above 100 mM until complete inhibition of digestion at 500 mM. Similarly, to SIN, GTL is bound to DNA preferentially within AT base pairs. In contrast to AITC, covalent modification of DNA by BITC was not observed in the tested concentration range. The aromatic substituent limited the reactivity of tested ITC to DNA.

Metabolic activation by S9 fraction caused a significant concentration-dependent increase in the ability of GTL to modify DNA (Fig. 8B); there was a clear inhibition of DNA digestion above 1 mM, and almost complete inhibition of digestion at 100 mM. Similarly, to SIN, there was also a change of the preferentially modified site to GC base pairs. In the case of BITC, similarly to AITC, a slight increase in chemical modification of GC base pairs in the concentration range from 1 to 100 mM was observed (Fig. 8B) suggesting that metabolic activation is needed to trigger reactivity of this aromatic ITC towards DNA.

Based on these results, it can be concluded that metabolic activation with S9 fraction, increased the ability of GLs to chemically modify DNA to a greater extent than their degradation products (AITC and BITC). The shift from AT specificity to GC specificity suggests that GLs tested were converted to other chemical forms whose reactivity resembled that of ITCs. Under cellular conditions, however, this DNA modification by GLs seems not to play any role, probably due to limited bioavailability, since neither of these compounds was cytotoxic or genotoxic towards HT29 cells.

#### Covalent DNA modifications by indole GL (GLB) and indolic degradation products

**Figure 9:**
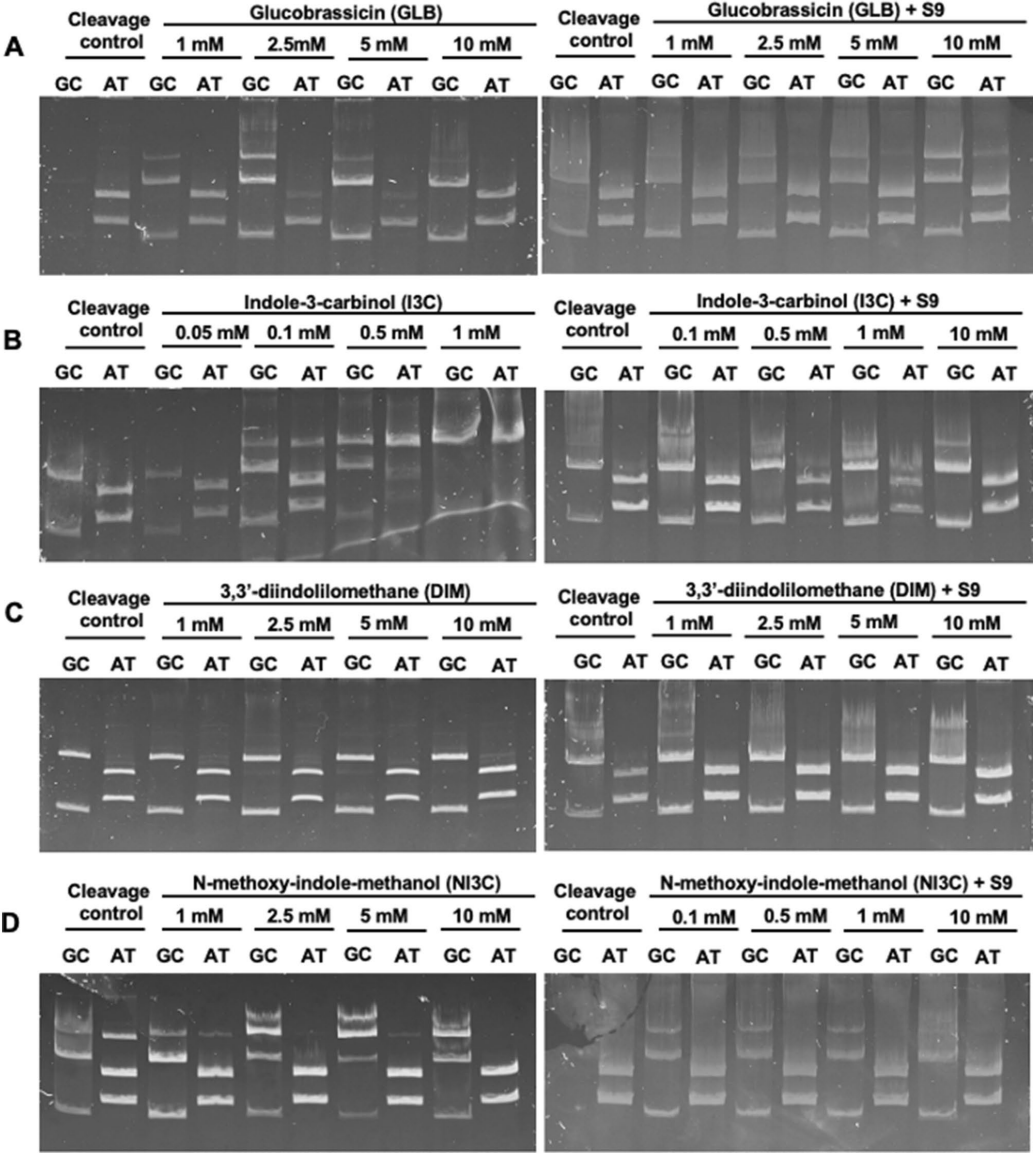
DNA fragments generated as a result of endonucleolytic cleavage of the DNA amplicon with HpaII (GC-specific) or Tru1I (AT-specific) enzymes. DNA amplicon incubated for 2 h at 37 °C with increasing glucobrassicin (GLB) (**A**), indole-3-carbinol (I3C) (**B**), 3,3′-diindolylmethane (DIM) (**C**), and *N*-methoxy-indole-3-carbinol (NI3C) (**D**) concentrations with and without metabolic activation with S9 fraction. After incubation restriction analysis was performed as described in the “Materials and methods” section. The control was the amplicon DNA incubated with water or DMSO in place of the test compound solution.

In the case of indolic compounds studied, in the tested range of concentrations (1–10 mM), only I3C and NI3C modified DNA amplicon, both preferentially at GC base pairs (Fig. 9A). Thus, the ability to form bonds with DNA occurred at much lower concentrations than it could be detected for aliphatic/aromatic counterparts (Figs. 7 and 8).

Metabolic activation with the S9 fraction (Fig. 9A) increased the reactivity of GBS and NI3C indoles towards DNA, but not that of I3C. Inhibition of DNA amplicon digestion by restriction endonucleases was noticed even at the lowest concentrations applied, preferentially at GC base pairs. The results are consistent with literature reports indicating the ability of indole GLs to covalently modify DNA^30,33^.

In contrast to other tested indoles, the metabolic activation of I3C with S9 microsomal fraction resulted in the virtually complete reversal of its ability to covalently modify DNA, and the apparent blur of the bands seemed to result rather from the interaction between the amplicon and S9 fraction proteins remaining in the mixture after extraction (Fig. 9B). This can be explained in two ways; either detoxification of intermediates capable of binding to DNA occurred or strong reactivity of the I3C caused its binding to the proteins present in the reaction mixture. However, in the latter case, the increase in DNA modification should be observed with the increasing concentration of this compound, which was not the case. Thus, it can be presumed that metabolism, as opposed to NI3C, contributes not to activation but detoxification of I3C. This would explain the higher cytotoxicity of NI3C compared to I3C towards cultured HT29 cells where metabolic conversion of indoles would be expected.

The results confirm the potential harmfulness of NI3C, already indicated by the previous studies^26,29,30,34^ according to which NI3C is mutagenic and exhibits significant ability to create DNA adducts in eukaryotic in vitro and in vivo models.

## Conclusions

Our study confirmed former claims that GLs show low biological activity due to their high polarity and thus limited bioavailability, and that the presence of an MYR enzyme is usually necessary for their activation. The only activity showed by all tested GLs was their ability to cause covalent modification of DNA. This ability increased after metabolic activation with enzymes of the S9 rat microsomal fraction, however, it was demonstrated only in a cell-free system.

GL hydrolysis products, mainly ITCs and indoles, are characterized by higher bioavailability than parent GLs and consequently, they exhibited stronger cytotoxic and genotoxic activities. In the case of ITCs (AITC and BITC), the increase in reactivity towards DNA was observed after incubation with the S9 fraction. I3C is readily bound to DNA, but metabolism by the S9 fraction resulted in the protective effect. Therefore, the harmfulness of this dietary compound seems negligible, at least in the model used and from the point of view of nucleic acids modification. Another situation occurs with NI3C, a product of neoglucobrassicin hydrolysis. The presence of the methoxy group decreased its reactivity towards DNA, but its metabolic activation with the S9 fraction increased it.

In general, our study demonstrated that GLs and their degradation products are dietary constituents displaying very different biological properties which are additionally modified by their structure, the presence of active MYR, and host metabolic activation system. Therefore, the current tendency to assess the biological activity of foods containing *Brassica* plants based solely on the content of GLs seems not appropriate.

## Materials and methods

### Chemicals and reagents

GLB was isolated from mature broccoli florets, broccoli sprouts, and pak choi sprouts in the German Institute of Human Nutrition, Potsdam-Rehbrϋcke^35^, *N*-methoxyindole-3-carbinol (NI3C) was synthesized in the same place^26^. Glucotropaeolin (GTL) was obtained from AppliChem GmbH (Darmstadt, Germany). Indole-3-acetonitrile (I3ACN) and indole-3-acetic acid (I3AA) were purchased from Merck (Darmstadt, Germany) and 3,3′-diindolylmethane (DIM), indole-3-carbinol (I3C), sinigrin (SIN), allyl isothiocyanate (AITC), benzyl isothiocyanate (BITC), myrosinase from *Sinapsis alba* (EC 3.2.1.147), sulfatase from *Helix pomatia* H1 (22,400 units/g solid) as well as reagents for electrophoresis were from Sigma-Aldrich (Taufkirchen, Germany). DNA amplicon was generated as reported before^31^. The molecular DNA weight standard was from DNA Gdansk (Poland), restriction endonucleases HpaII, Tru1I and buffers Tango^®^/R^®^ from Fermentas (Lithuania), nucleic acid gel stain SYBR^®^ Gold from Molecular Probes (Eugene, OR, USA), nucleic acid gel stain Gel Green^®^ from Biotium (Fremont, CA, USA) and MTT, cell culture McCoy's 5A medium, fetal bovine serum and streptomycin-penicillin were from Sigma-Aldrich. Not listed chemicals and biochemicals were purchased from Sigma-Aldrich and were of the appropriate grade.

### Cell culture

Human colon adenocarcinoma (HT29) cells (ATCC) grown in a Smart cell incubator (Heal Force, 37 °C, 5% CO_2_). McCoy’s growth medium supplemented with antibiotics (Penicillin 100 U/mL and Streptomycin 100 mg/mL) and foetal bovine serum (10%, v/v) was used for cell culture. Cells were regularly checked for mycoplasma contamination (Universal Mycoplasma Detection Kit, ATCC, Manassas, VA, USA).

### Antiproliferative activity (MTT test)

For the determination of the antiproliferative activity of tested compounds, the MTT test was employed using the procedure described before^36^. About 15,000 cells in 0.15 mL medium were seeded in 96-well tissue culture plates and left to settle for 24 h at 37 °C. Then, the cells were incubated for 3, 6, 24 and 72 h with different concentrations of the test compounds (GL or ITC/indole) or with the mixture of GLs and enzyme MYR (1 mU of MYR per 1 nmol of GL). After 72 h 0.05 mL of MTT solution (4 mg/mL) was added and again cells were incubated for 4 h. After this time medium was removed from the formazan crystals and they were dissolved in DMSO. The absorption was measured at 540 nm with TECAN Infinite M200 plate reader (Tecan Group Ltd, Switzerland). The antiproliferative effect was presented as growth inhibition of treated cells compared to control (non-treated) cells. Results were also expressed as Accumulated Survival Index (ASI), calculated as the sum of areas under survival curves for each exposure time for the same concentration range.

### Genotoxic activity (comet assay)

The genotoxic activity of GLs and their degradation products was determined by the comet assay. Briefly, human colon cancer (HT29) cells were seeded in 24-well plates (200,000 cells per well in 1.8 mL of medium). After 24 h of incubation, cells were treated with 0.2 mL of the test compounds (GL or ITC/indole) or with the mixture of GLs with the enzyme myrosinase (MYR; 1 mU of MYR per 1 nmol of GL) for 16 h (37 °C, 5% CO_2_). Based on MTT assay results, non-cytotoxic concentrations of the test compounds were selected. As negative control served cells treated with PBS and as positive control cells treated with 450 μM H_2_O_2_. After exposure, cells were detached, resuspended in 2 mL of fresh medium and comet assay was carried out according to the previously described protocol^18^. The cell nuclei after single-cell electrophoresis embedded on microscope slides were stained with fluorescent dye Gel Green^®^ for 20 min at 4 °C and comets were analyzed using a fluorescence microscope (Zeiss, Germany) equipped with Metafer4^®^ software. The results are presented as the mean % of DNA in tail determined in 200 consecutive comets per gel (2 gels per microscope slide).

### Covalent DNA modification in a cell-free system (restriction analysis)

Restriction analysis technique was used to determine the ability of tested compounds to modify DNA in cell-free system as described before^31^. DNA amplicon with two restriction sites (GC site recognized by enzyme HpaII and AT site recognized by Tru1I) is used in this method. This amplicon may be potentially modified and as a result the cleavage by these enzymes will be inhibited. Briefly, DNA amplicon was incubated for 2 h at 37 °C, with the solution of tested GLs, their hydrolysis products or mixture of GL and MYR. For negative control water or DMSO were used. For metabolic activation rat liver S9 microsomal fraction was used. After the treatment, samples were extracted using chloroform/isoamyl alcohol as described before^31^. Next, the restriction analysis was performed using HpaII or Tru1I endonucleases (4 h, 37 °C). To analyse obtained DNA fragments, electrophoresis in polyacrylamide gel in non-denaturing conditions was performed (6% polyacrylamide gel, 90 min, 90 V, TBE buffer). DNA bands were stained using SYBR Gold solution for 20 min at 4 °C and visualized with UV transilluminator. At this point, gels were photographed using a digital camera (Canon EOS 1000D).

### Statistical analysis

All values represent means ± SD of three independent experiments. Statistical analysis was performed using one-way analysis of variance (ANOVA) with Prism 4 (Version 4.0c, GraphPad Software Inc.).

## Supplementary Information

Supplementary Information 1.

Supplementary Information 2.

Supplementary Information 3.

Supplementary Information 4.

Supplementary Information 5.

Supplementary Information 6.

Supplementary Information 7.

Supplementary Information 8.

Supplementary Information 9.

Supplementary Information 10.

Supplementary Information 11.

Supplementary Information 12.

Supplementary Information 13.

Supplementary Information 14.

Supplementary Information 15.

Supplementary Information 16.

## Abbreviations

2AA: 2-Aminoanthracene
2NF: 3-Nitrofluorene
4-NQO: 4-Nitroquinoline *N*-oxide
AITC: Allyl isothiocyanate
BITC: Benzyl isothiocyanate
DIM: 3,3′-Diindolylmethane
DMSO: Dimethyl sulfoxide
GLB: Glucobrassicin
GLs: Glucosinolates
GRE: Glucoraphanin
GRH: Glucoraphasatin
GSNT: Gluconasturtin
GTL: Glucotropaeolin
I3AA: Indole-3-acetic acid
I3ACN: Indole-3-acetonitrile
I3C: Indole-3-carbinol
ITCs: Isothiocyanates
MTT: Thiazozyl blue tetrazolium bromide
MYR: Myrosinase
neoGLB: Neoglucobrassicin
NI3C: *N*-Methoxy-indole-3-carbinol
Nrf2: Nuclear factor erythroid 2
PBS: Phosphate-buffered saline
SIN: Sinigrin
SULT: Sulfotransferase
