# Supplementary figures and images for: The comparison of cytotoxic and genotoxic activities of glucosinolates, isothiocyanates, and indoles
Source: Sci Rep. 2022 Mar 22;12:4875. doi: 10.1038/s41598-022-08893-8 (PMC8940953; doi:10.1038/s41598-022-08893-8)

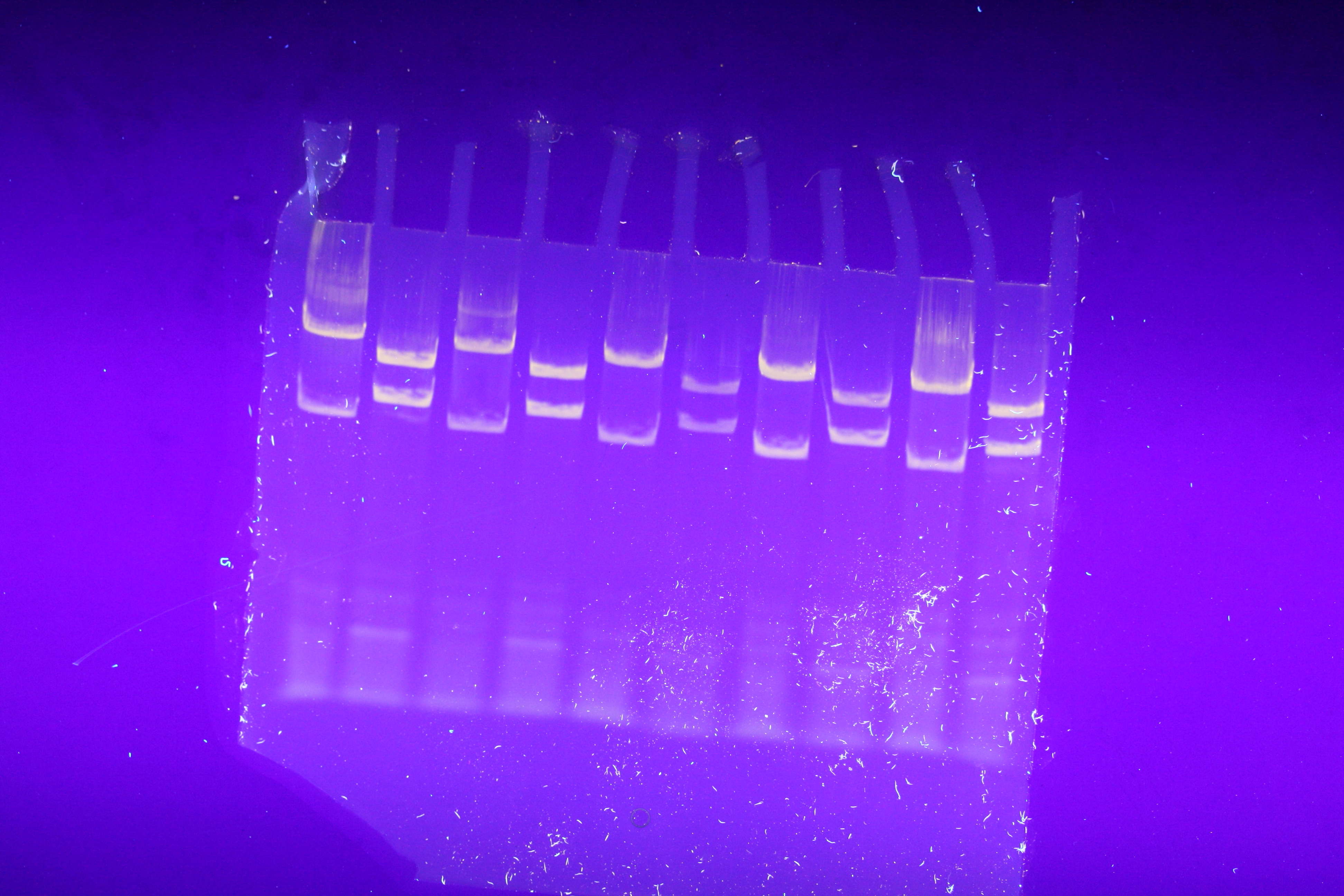

Supplement: Supplementary file 1 — Supplementary Information 1. [file 41598_2022_8893_MOESM1_ESM.jpg]

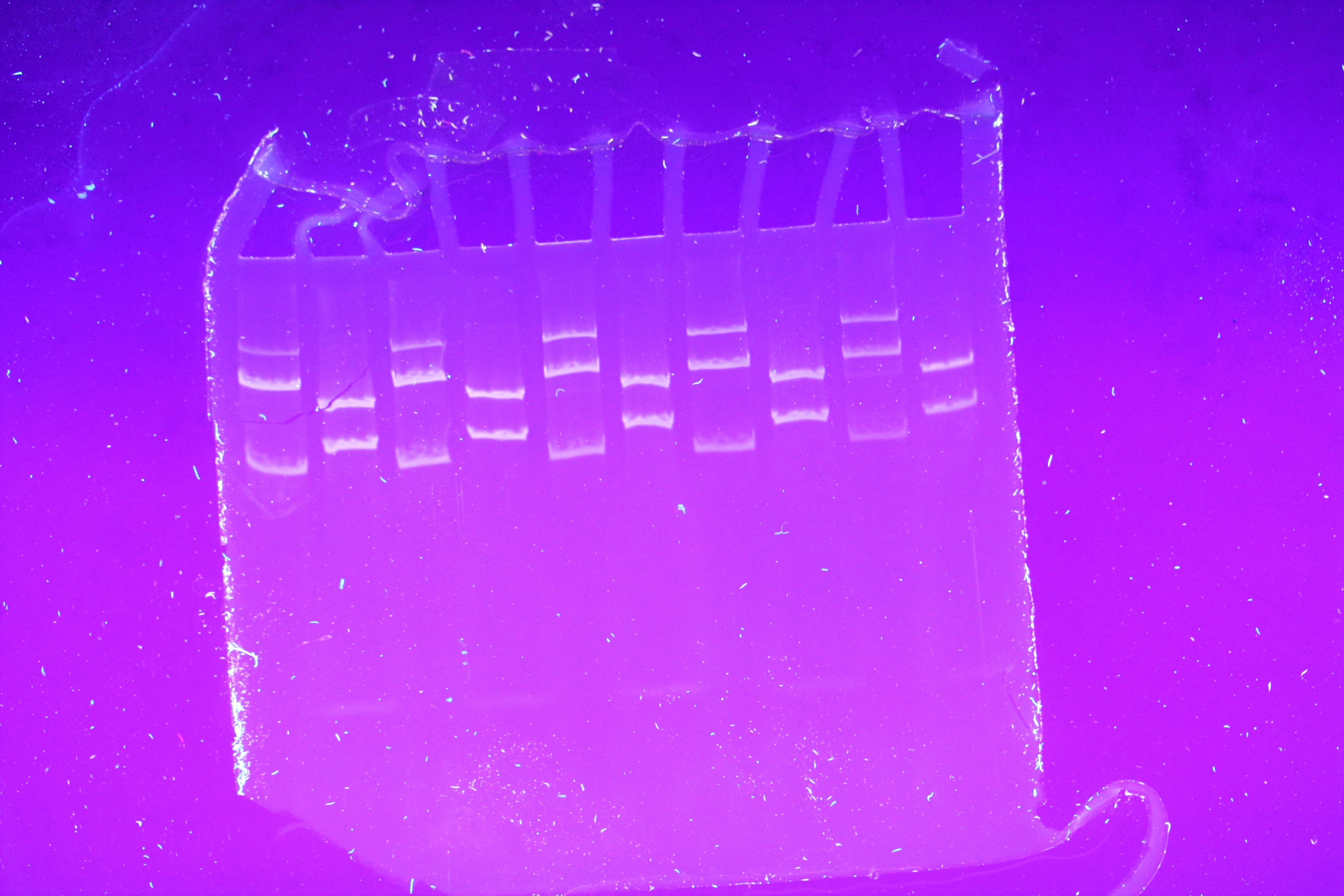

Supplement: Supplementary file 2 — Supplementary Information 2. [file 41598_2022_8893_MOESM2_ESM.jpg]

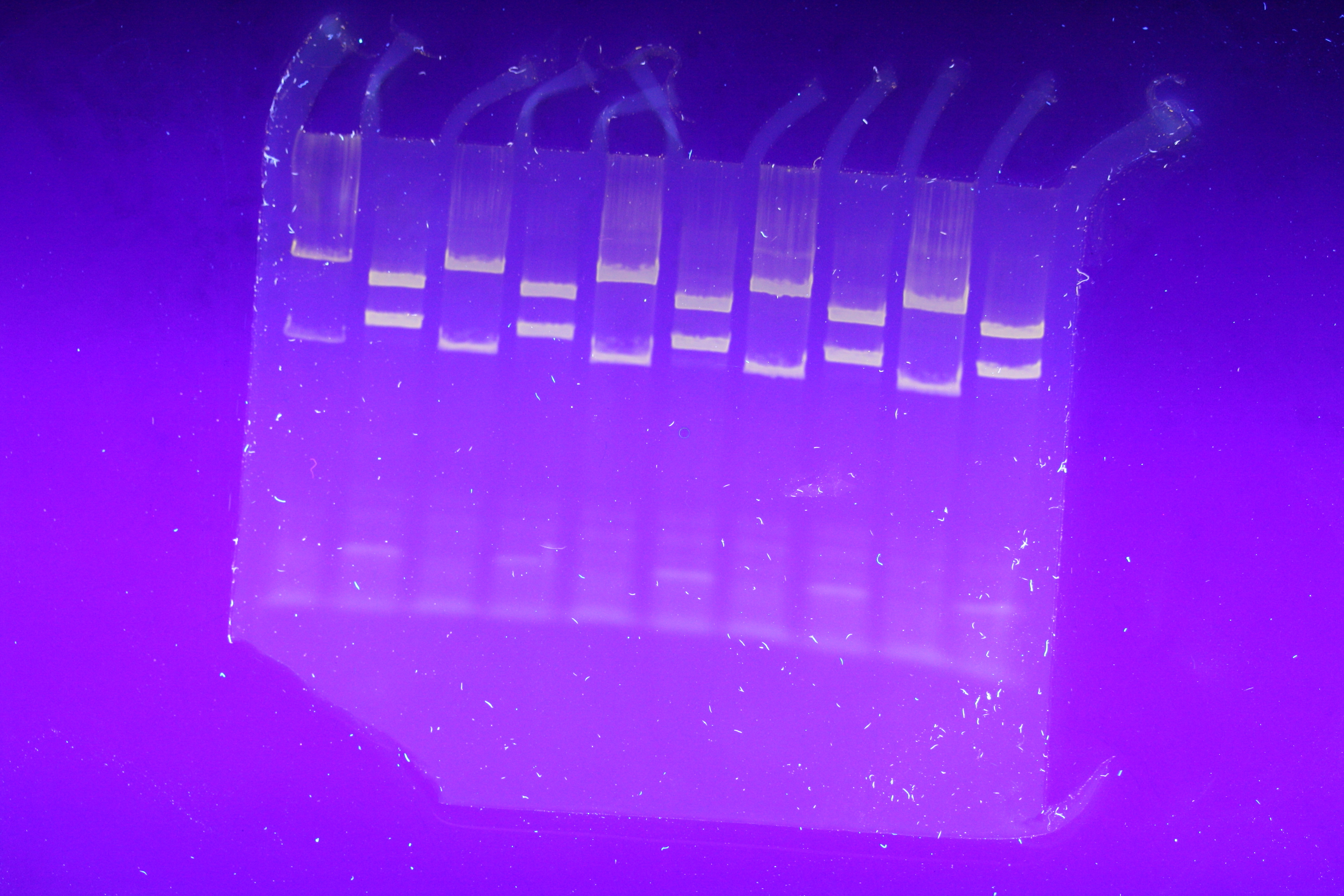

Supplement: Supplementary file 3 — Supplementary Information 3. [file 41598_2022_8893_MOESM3_ESM.jpg]

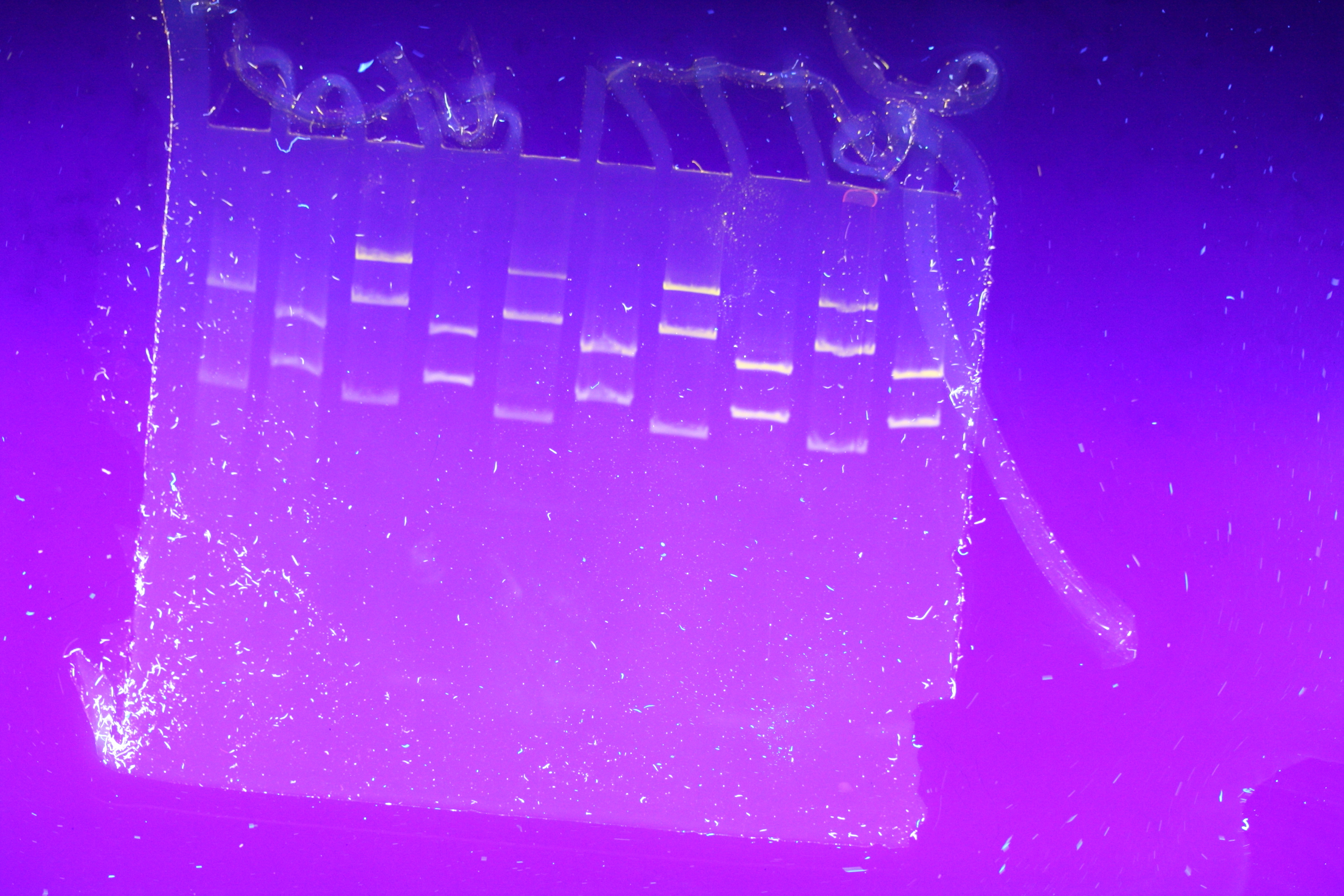

Supplement: Supplementary file 4 — Supplementary Information 4. [file 41598_2022_8893_MOESM4_ESM.jpg]

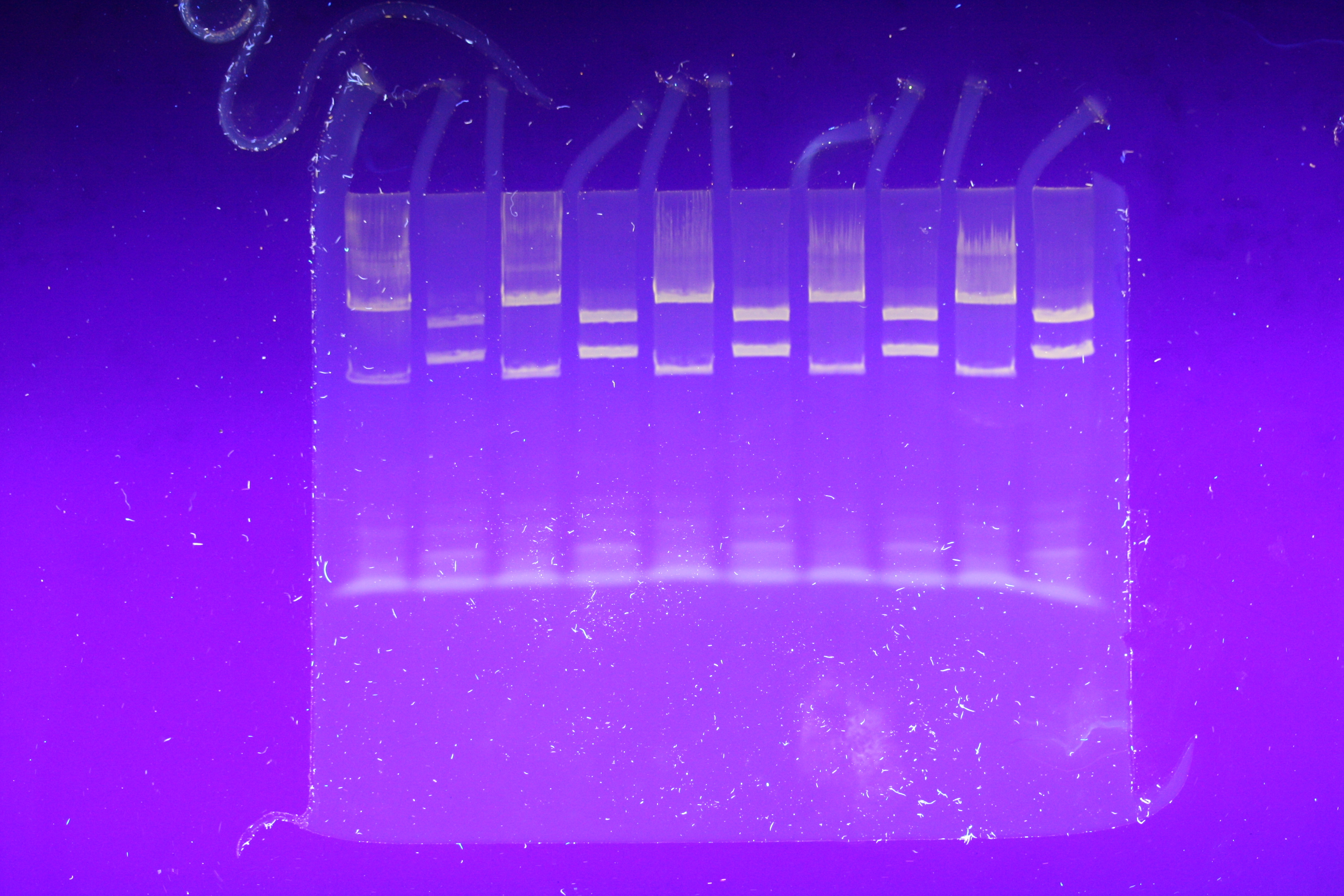

Supplement: Supplementary file 5 — Supplementary Information 5. [file 41598_2022_8893_MOESM5_ESM.jpg]

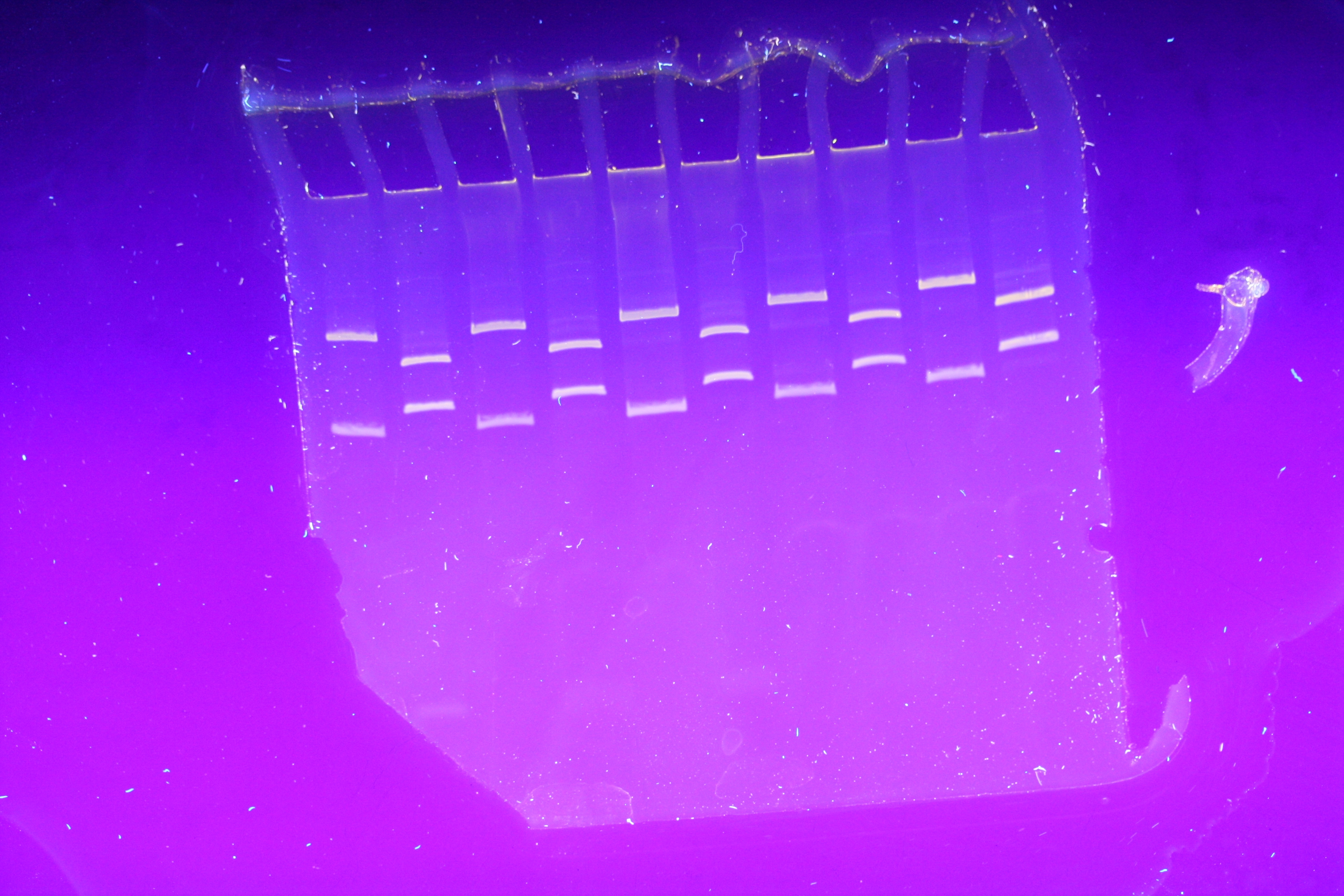

Supplement: Supplementary file 6 — Supplementary Information 6. [file 41598_2022_8893_MOESM6_ESM.jpg]

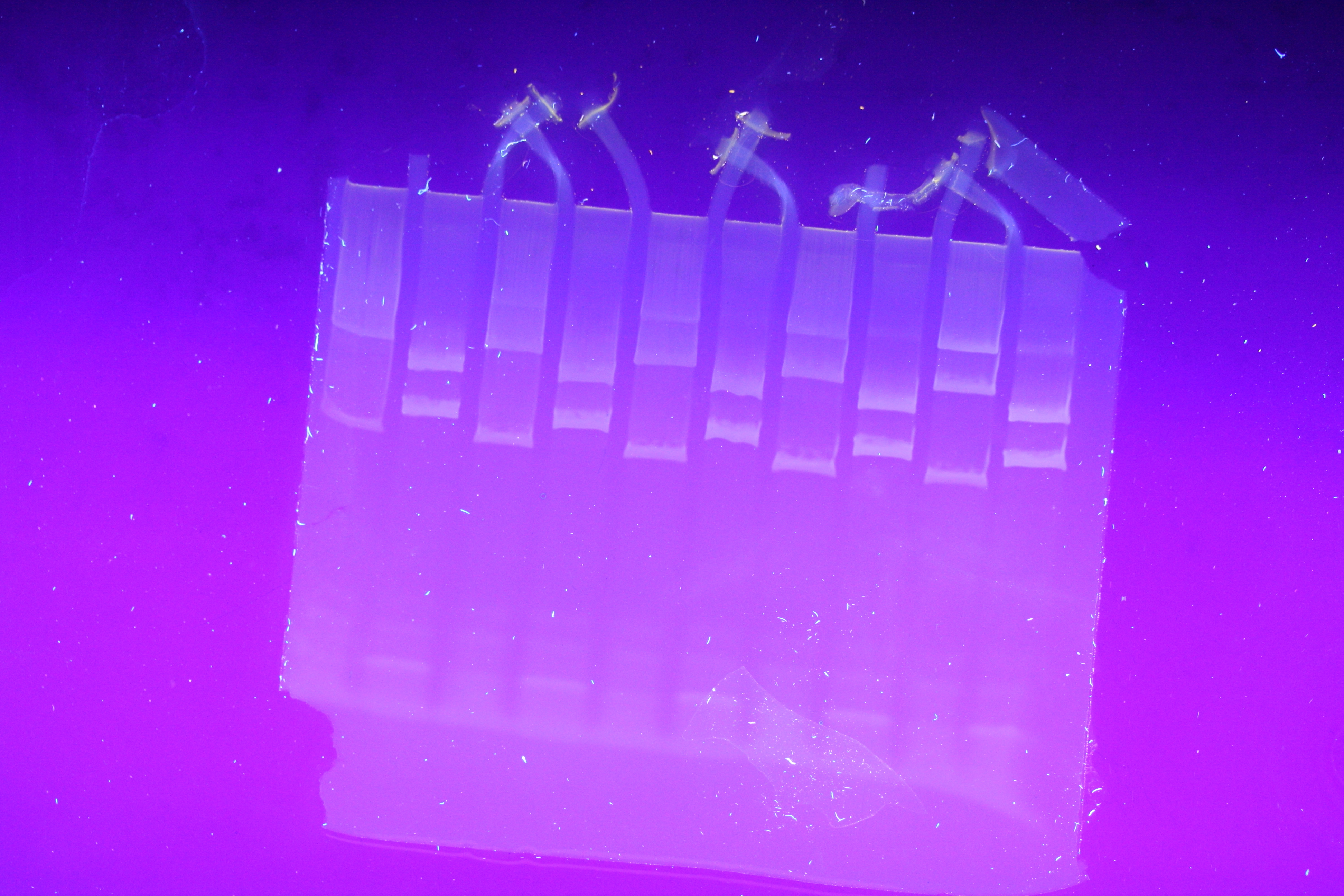

Supplement: Supplementary file 7 — Supplementary Information 7. [file 41598_2022_8893_MOESM7_ESM.jpg]

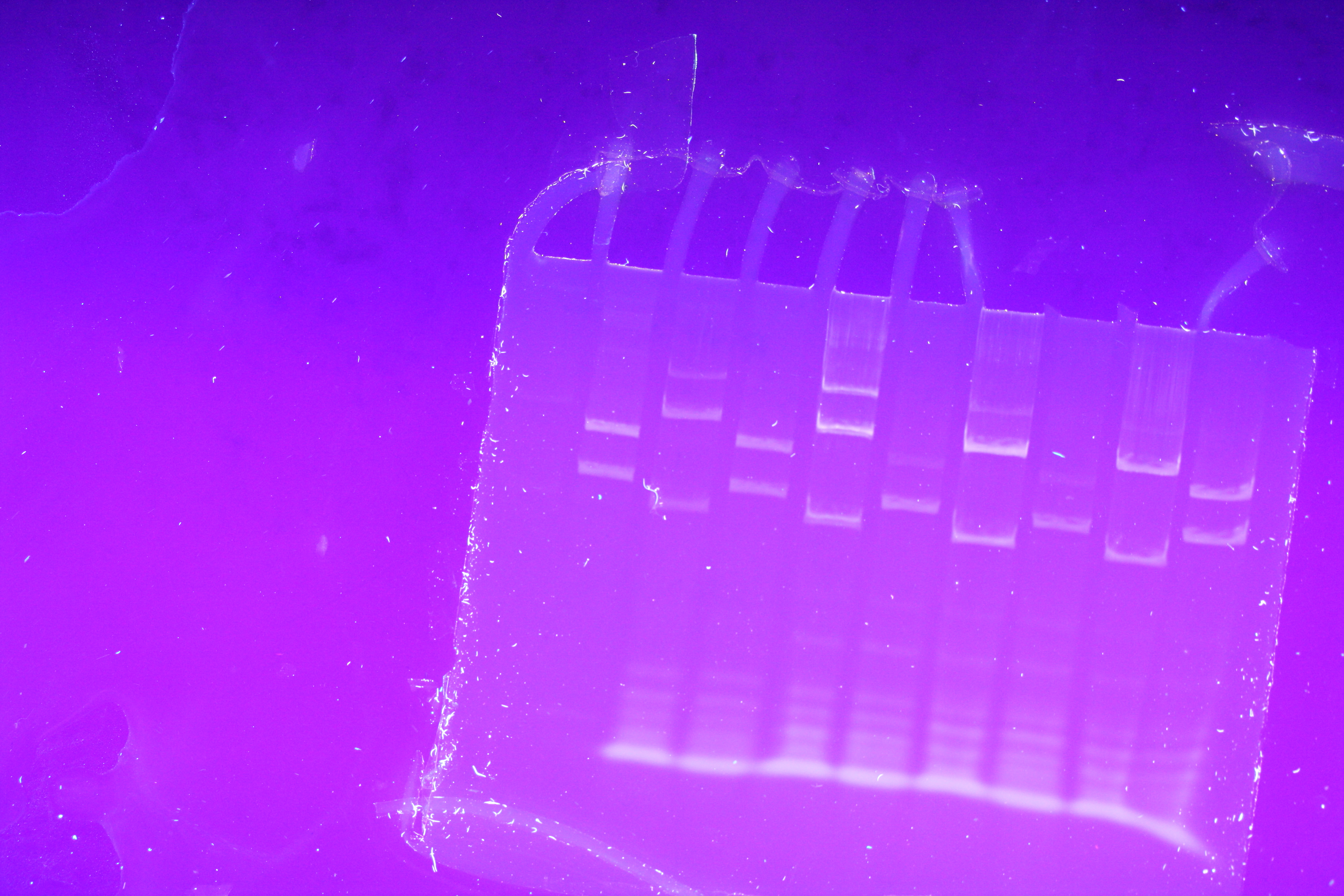

Supplement: Supplementary file 8 — Supplementary Information 8. [file 41598_2022_8893_MOESM8_ESM.jpg]

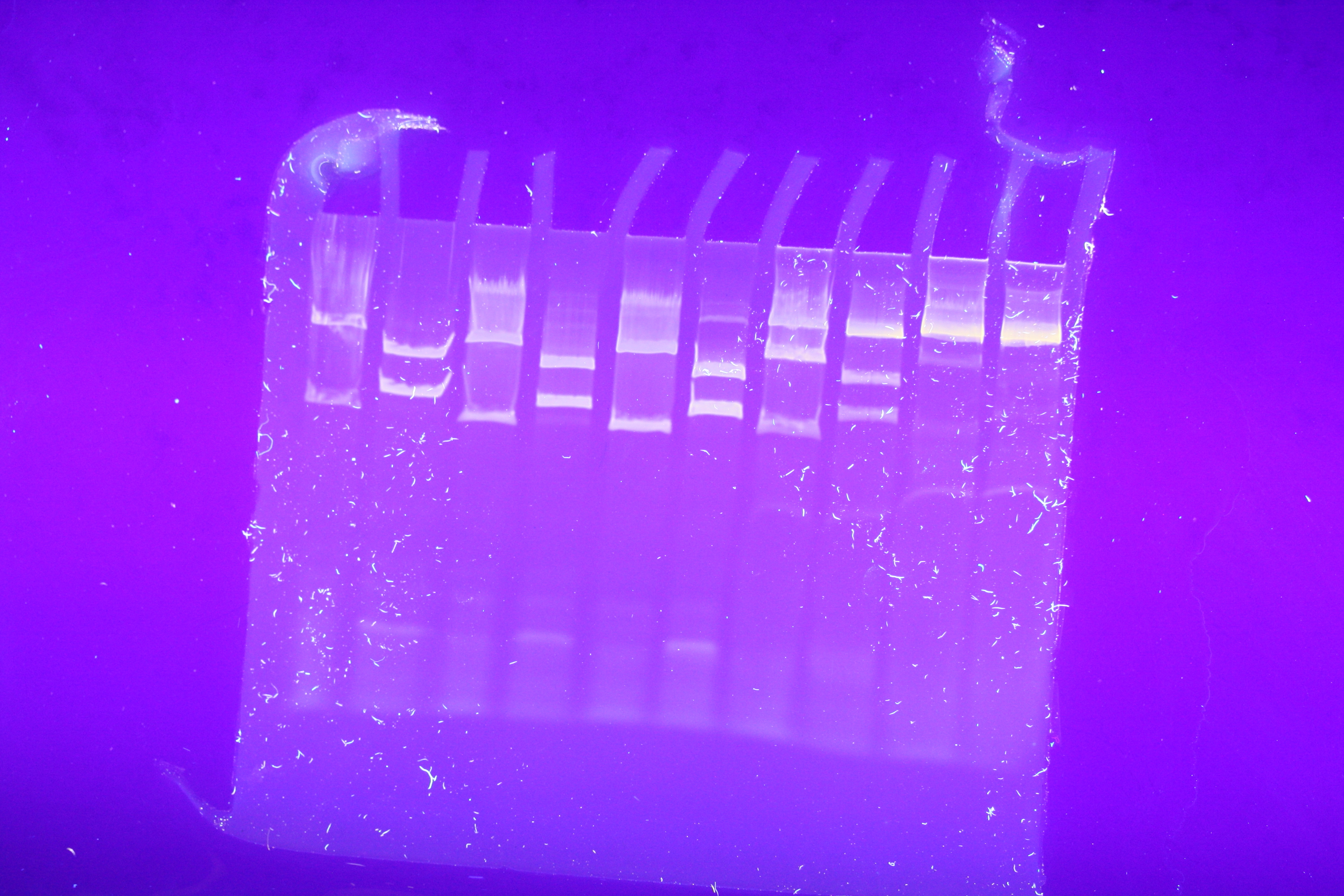

Supplement: Supplementary file 9 — Supplementary Information 9. [file 41598_2022_8893_MOESM9_ESM.jpg]

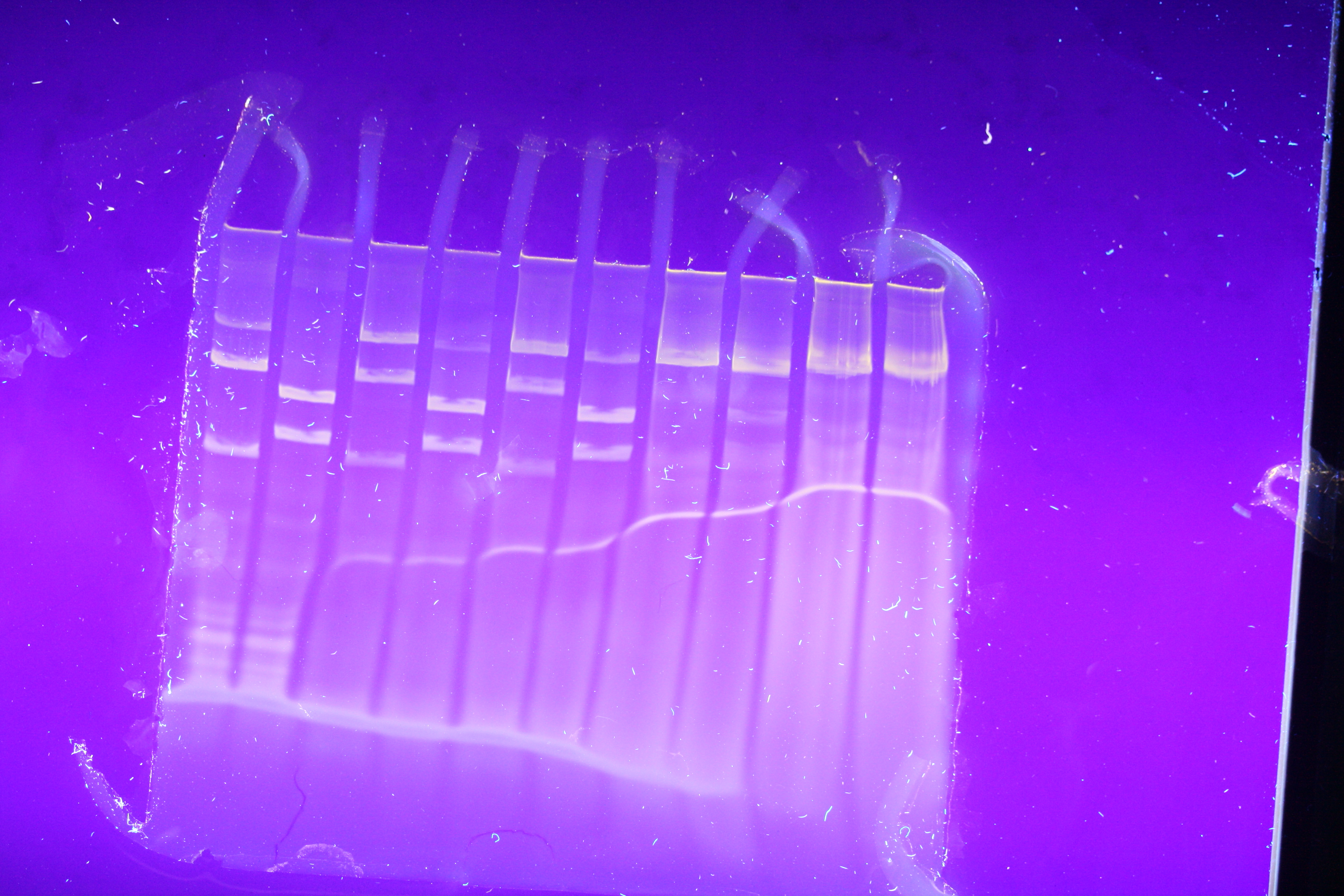

Supplement: Supplementary file 10 — Supplementary Information 10. [file 41598_2022_8893_MOESM10_ESM.jpg]

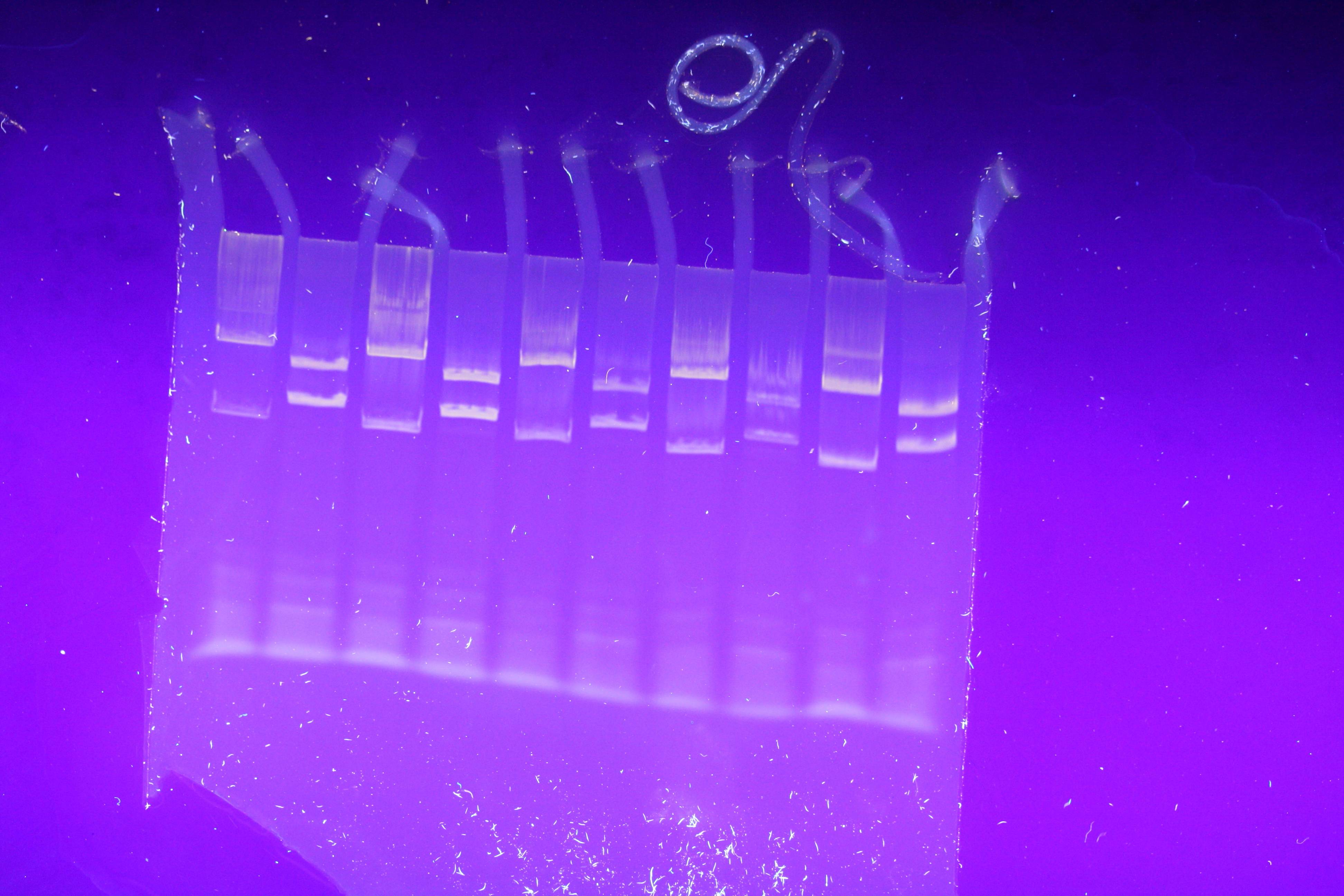

Supplement: Supplementary file 11 — Supplementary Information 11. [file 41598_2022_8893_MOESM11_ESM.jpg]

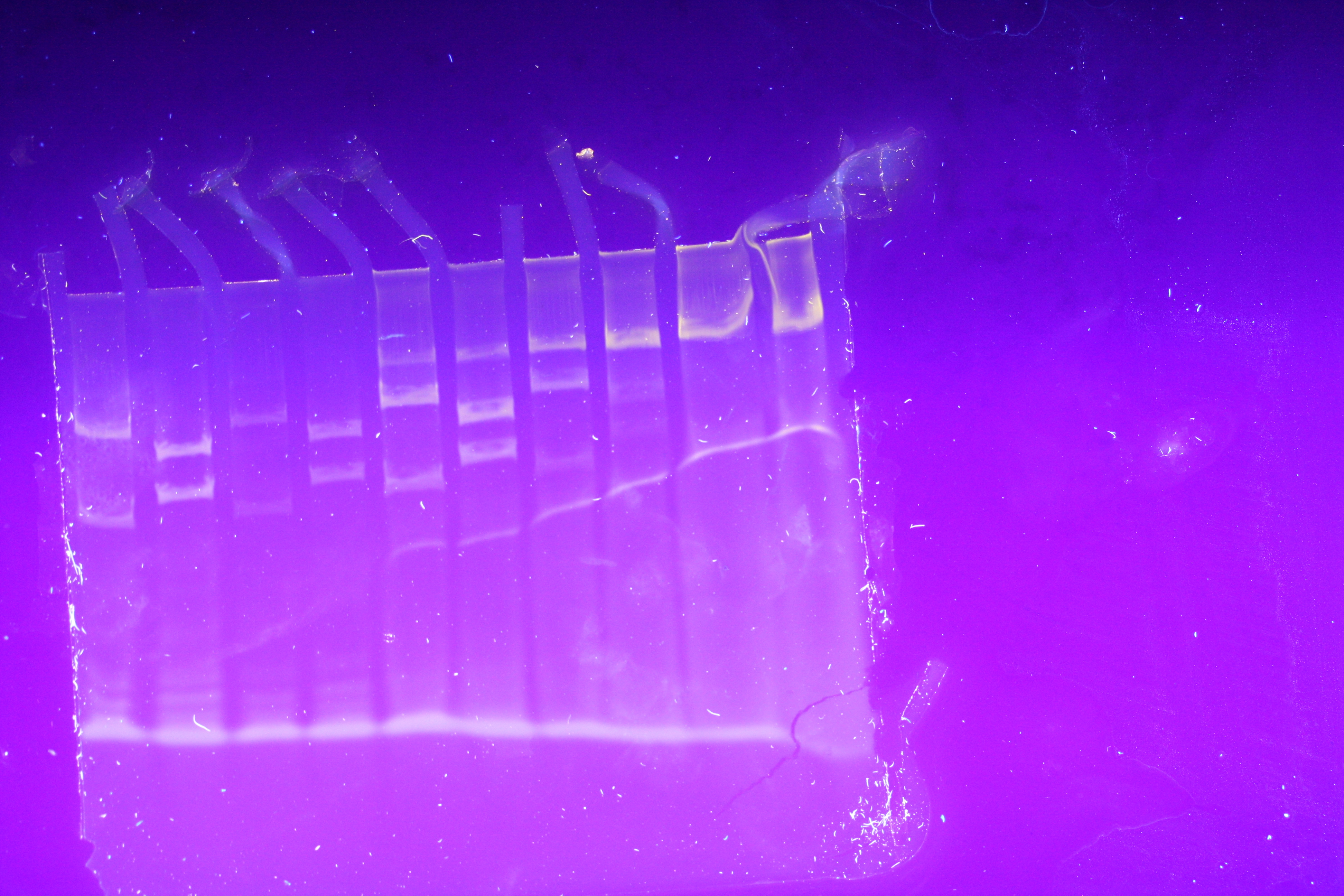

Supplement: Supplementary file 12 — Supplementary Information 12. [file 41598_2022_8893_MOESM12_ESM.jpg]

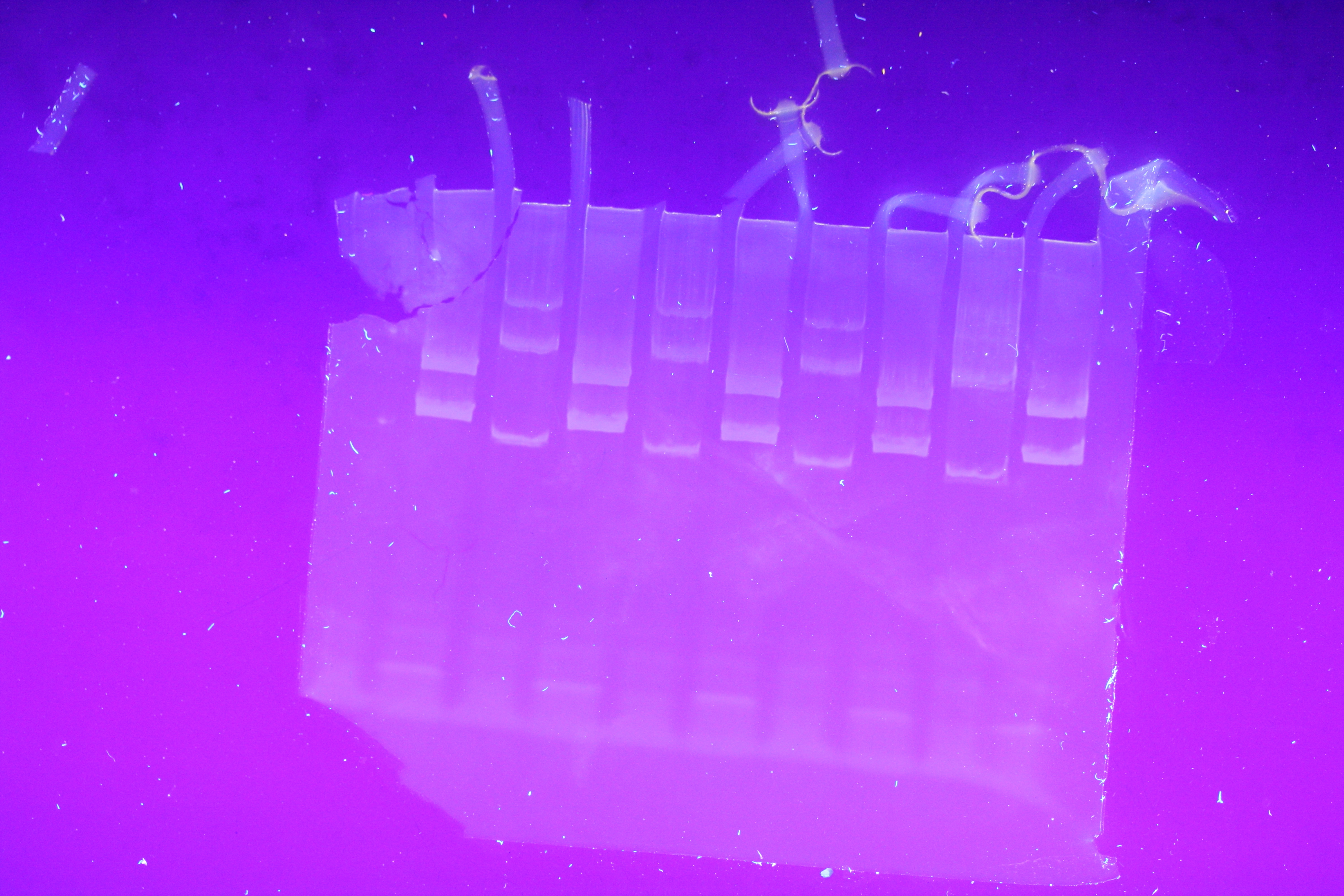

Supplement: Supplementary file 13 — Supplementary Information 13. [file 41598_2022_8893_MOESM13_ESM.jpg]

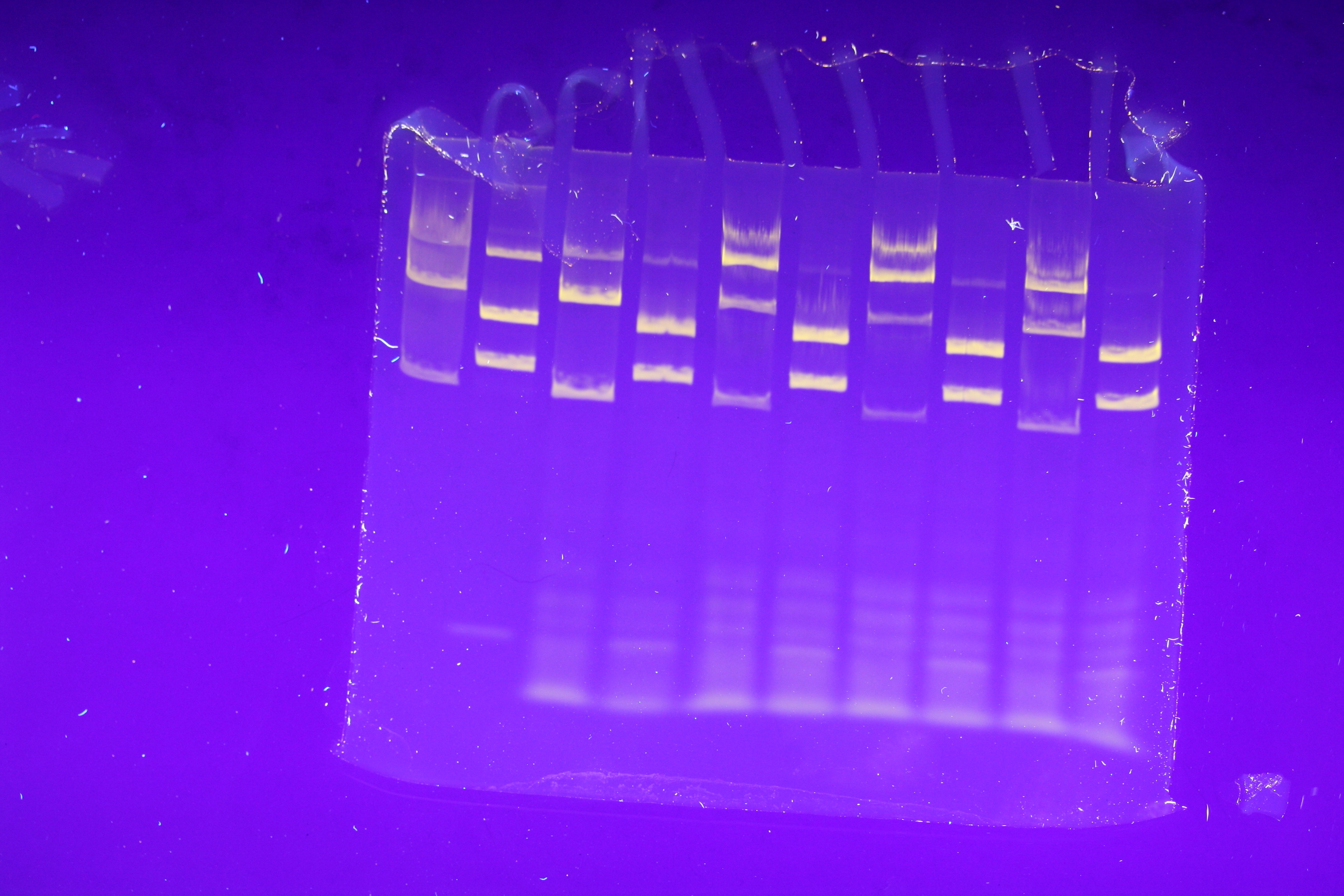

Supplement: Supplementary file 14 — Supplementary Information 14. [file 41598_2022_8893_MOESM14_ESM.jpg]

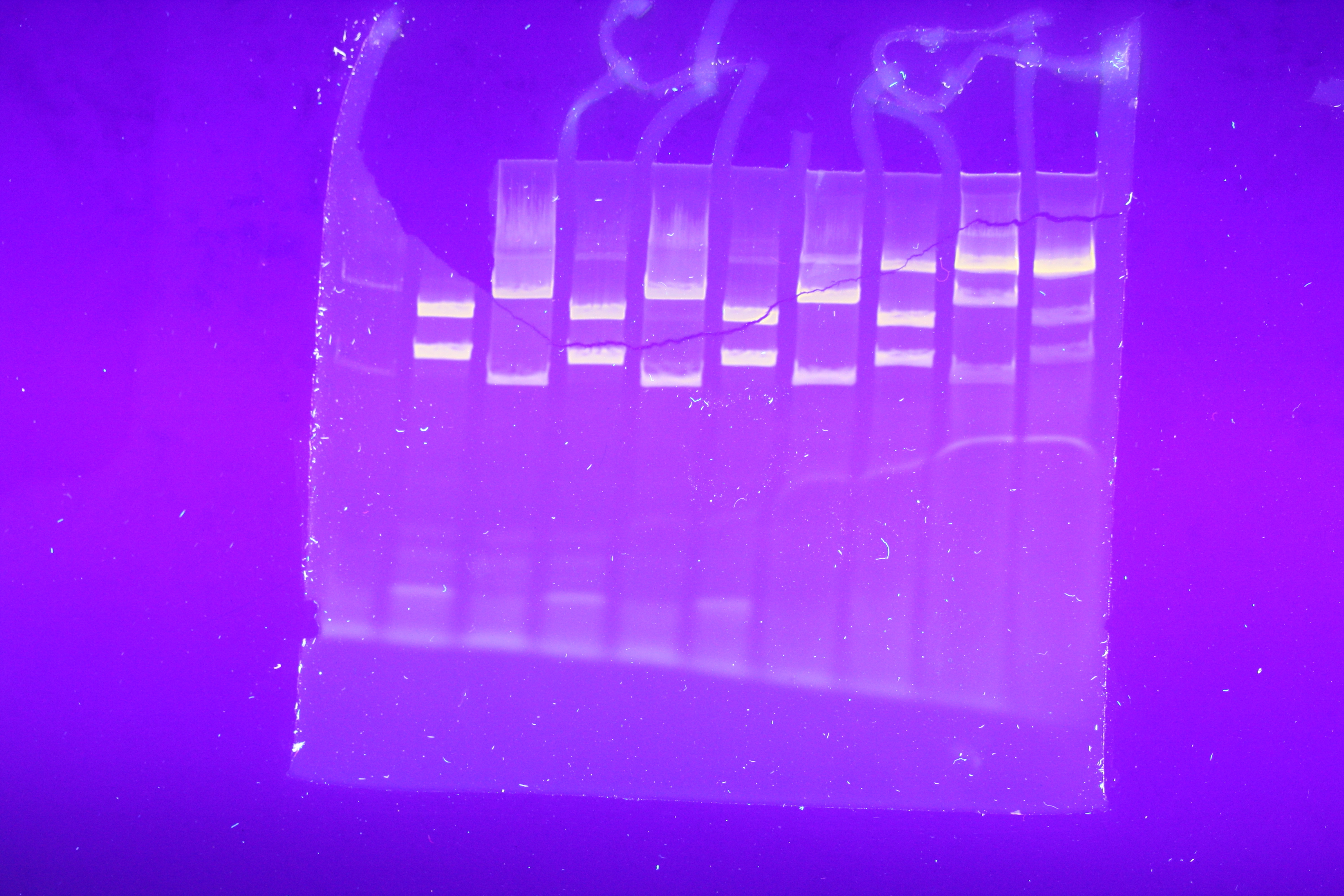

Supplement: Supplementary file 15 — Supplementary Information 15. [file 41598_2022_8893_MOESM15_ESM.jpg]

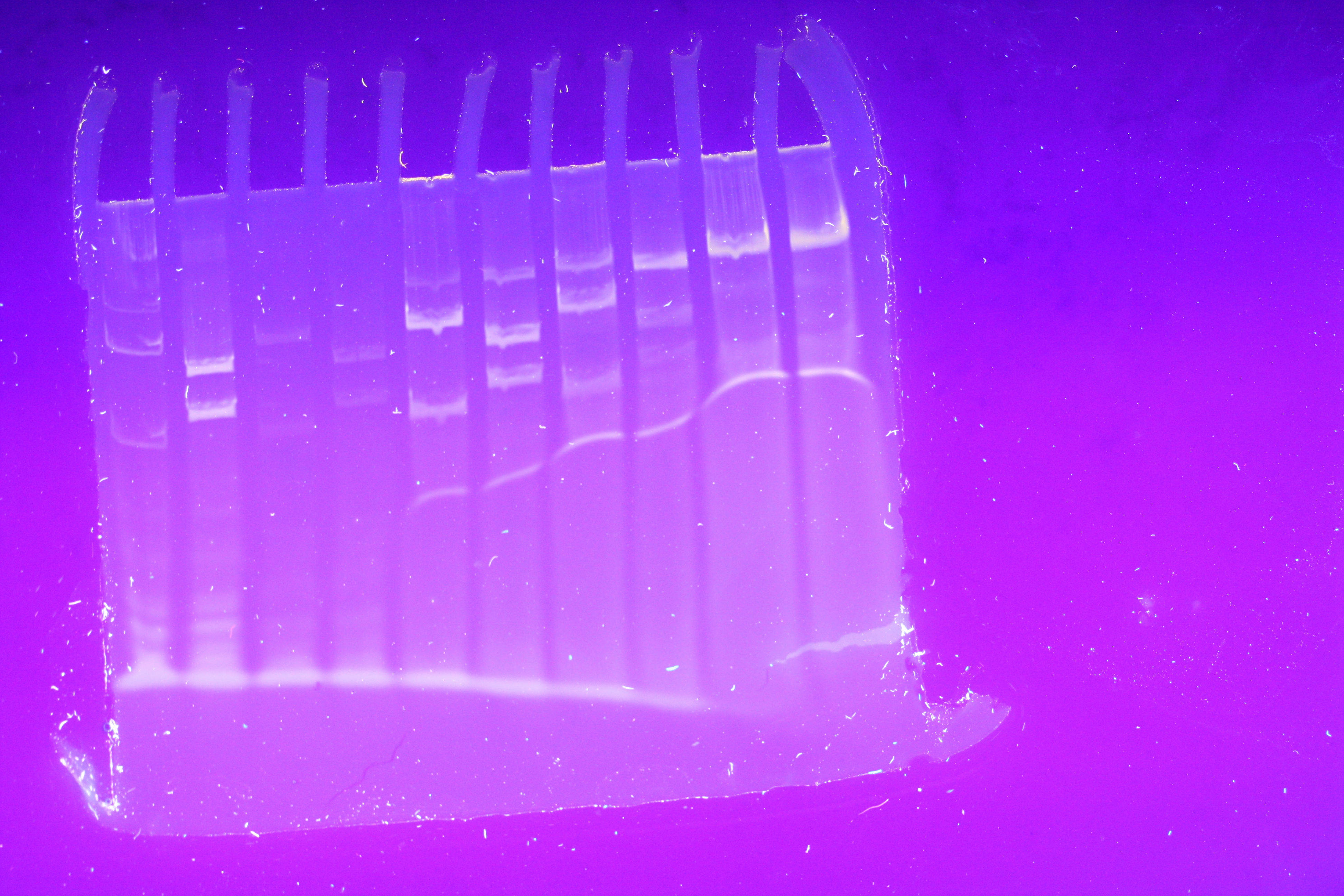

Supplement: Supplementary file 16 — Supplementary Information 16. [file 41598_2022_8893_MOESM16_ESM.jpg]
